# Axial Oxygen Ligands Regulating Electronic and Geometric Structure of Zn‐N‐C Sites to Boost Oxygen Reduction Reaction

**DOI:** 10.1002/advs.202302152

**Published:** 2023-06-26

**Authors:** Qiuyan Jin, Chenhui Wang, Yingying Guo, Yuhang Xiao, Xiaohong Tan, Jianpo Chen, Weidong He, Yan Li, Hao Cui, Chengxin Wang

**Affiliations:** ^1^ School of Materials Science and Engineering Sun Yat‐sen University Guangzhou 510275 China; ^2^ The Key Laboratory of Low‐Carbon Chemistry & Energy Conservation of Guangdong Province Sun Yat‐sen University Guangzhou 510275 China

**Keywords:** axial ligands engineering, *d*‐orbital regulation, oxygen reduction reaction, single atom catalyst, zinc‐air battery

## Abstract

Zn‐N‐C possesses the intrinsic inertia for Fenton‐like reaction and can retain robust durability in harsh circumstance, but it is often neglected in oxygen reduction reaction (ORR) because of its poor catalytic activity. Zn is of fully filled 3d^10^4s^2^ configuration and is prone to evaporation, making it difficult to regulate the electronic and geometric structure of Zn center. Here, guided by theoretical calculations, five‐fold coordinated single‐atom Zn sites with four in‐plane N ligands is constructed and one axial O ligand (Zn‐N_4_‐O) by ionic liquid‐assisted molten salt template method. Additional axial O not only triggers a geometry transformation from the planar structure of Zn‐N_4_ to the non‐planar structure of Zn‐N_4_‐O, but also induces the electron transfer from Zn center to neighboring atoms and lower the *d*‐band center of Zn atom, which weakens the adsorption strength of *OH and decreases the energy barrier of rate determining step of ORR. Consequently, the Zn‐N_4_‐O sites exhibit improved ORR activity and excellent methanol tolerance with long‐term durability. The Zn‐air battery assembled by Zn‐N_4_‐O presents a maximum power density of 182 mW cm^−2^ and can operate continuously for over 160 h. This work provides new insights into the design of Zn‐based single atom catalysts through axial coordination engineering.

## Graphical abstract

Axial O ligand engineering triggers a geometry transformation from the planar structure of Zn‐N_4_ to the non‐planar structure of Zn‐N_4_‐O and tailors the electronic structure of central Zn site. As a result, Zn‐N_4_‐O SACs deliver the significantly promoted ORR activity with the half‐wave potential of 0.884 V in 0.1 m KOH.

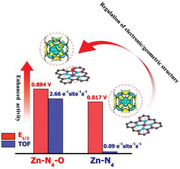

## 1 Introduction

Oxygen reduction reaction (ORR) as a fundamental and crucial half‐reaction plays a key role in diverse green energy conversion and storage devices, including metal‐air batteries and fuel cells.^[^ ^1^, ^2^, ^3^, ^4^, ^5^ ^]^ Pt‐group materials are recognized as highly active electrocatalysts to boost sluggish kinetics of ORR process, whereas, the earth scarcity and unsatisfied long‐term durability restrict wide applications of them.^[^ ^6^, ^7^, ^8^ ^]^ Developing cost‐efficient none‐noble catalysts with competitive and stable activity has attracted wide attentions.^[^ ^9^, ^10^, ^11^, ^12^, ^13^, ^14^, ^15^ ^]^ Recently, single atom catalysts (SACs) with atomic metal‐N_x_ (M‐N_x_) moieties have been regarded as promising alternatives to Pt due to their desired ORR activity, maximum utilization of metal sites and tunable coordination environment of central metal.^[^ ^16^, ^17^ ^]^

So far, various non‐precious metal transition metals, such as Fe, Co, Ni, Mn, etc., have been reported as central metal sites for SACs catalysts.^[^ ^18^ ^]^ In contrast to above metals, Zn have been rarely explored to construct SACs due to its poor ORR activity. In fact, Zn SACs possesses some natural advantages in ORR due to the fully occupied 3d10 configuration of Zn atom. Zn‐N‐C is inactive for Fenton‐reaction, which would reduce the damage to electrode and electrolyte membrane. Moreover, Zn have promising environmental compatibility, which can retain robust durability in harsh circumstance.^[^ ^19^, ^20^ ^]^ Accordingly, some reports have focused on improving the ORR activity of Zn SACs. For instance, Li et al. synthesized ultrahigh‐loading Zn single‐atom catalysts to improve ORR performance by strictly controlling the gasification rate of Zn precursor.^[^ ^19^ ^]^ However, the poor intrinsic ORR activity of Zn center remains unresolved. This inspires some investigations to improve its intrinsic catalytic activity. Wang et al. regulated the in‐plane coordination environment of Zn sites via introducing boron species and the obtained N_2_‐Zn‐B_2_ structure was confirmed as the contributing center for ORR process.^[^ ^20^ ^]^ Zhang et al. presented that adjusting the Zn neighboring planar N/C coordination ratio can distinctly alter electronic state and thus facilitate the ORR process.^[^ ^21^ ^]^ Despite these encouraging efforts to regulate Zn central coordinated environment, the electronic structure of Zn site is insensitive to changes in planar local microenvironment due to the fully filled 3d^10^ configuration. Interestingly, recent studies found that the introduction of the axial coordination can break the electronic distribution symmetry of center metal and directly regulate the electronic property of d orbital,^[^ ^22^, ^23^ ^]^ further achieving the optimization of catalytic activity, selectivity and durability.^[^ ^24^, ^25^ ^]^ Wang and co‐workers indicated that axial Co‐O coordination was able to induce the electronic delocalization of Co center, thus optimizing the intermediate adsorption and improving ORR activity.^[^ ^26^ ^]^ Chen et al. presented that the axial coordinated O atoms can distinctively regulate the electrons in the Fe 3d orbitals, even better than that of axial N.^[^ ^27^ ^]^ Compared with other metals, Zn is of fully filled 3d^10^4s^2^ configuration and is prone to evaporation, making it difficult to bind additional axial ligands to Zn‐N‐C sites. It is very attractive to probe the influence of axial ligand on the local coordination geometry/electron configuration of Zn centers and to clarify the corresponding structure‐activity relationships.

Hereby, we propose a strategy engendered by the axial coordinated O to improve the intrinsic activity of ZnN_4_ toward ORR. First, the possible structure models and corresponding ORR performance of Zn‐N_4_‐O are discussed theoretically. The calculation results show that the axial O coordination site can induce the electron transfer from Zn center to neighboring atoms and achieve electronic delocalization, which can optimize the adsorption strength of *OH and lower the energy barrier of rate determining step (RDS), resulting in enhanced ORR performance. As proof of concept, an ionic liquid‐assisted molten salt template strategy is developed to fabricate five‐fold coordinated Zn SACs, consisting of four in‐plane nitrogen ligands and one axial direction O ligand (Zn‐N_4_‐O). In line with the theoretical calculations, axial O ligand engineering triggers a geometry transformation from the planar structure of Zn‐N_4_ to the non‐planar structure of Zn‐N_4_‐O, and thus tailors the electronic structure of central Zn site. As a result, Zn‐N_4_‐O SACs deliver the significantly promoted ORR activity with the half‐wave potential of 0.884 V in 0.1 m KOH, which are superior to those of Zn‐N_4_ SACs (0.817 V) and commercial Pt/C (0.855 V). More importantly, the Zn‐N_4_‐O SACs present high intrinsic ORR activity with superior turn‐over frequency (2.66 e^−1^ site^−1^ s^−1^), which is orders of magnitude higher than that of Zn‐N4 (0.09 e^−1^ site^−1^ s^−1^). Meanwhile, natural advantages of robust stability and inactive Fenton‐reaction are well retained, and no obvious degeneration can be observed on ORR activity of Zn‐N_4_‐O after undergoing 10 000 cycles accelerated durability test. The assembled Zn‐air battery offers a peak power density of 182 mW cm^−2^, and that can operate continuously for least 160 h, highlighting the ultra‐stable feature of Zn‐N_4_‐O and promise potential in application for energy device.

## 2 Results and Discussions

### 2.1 Theoretical Predictions

**Figure 1:**
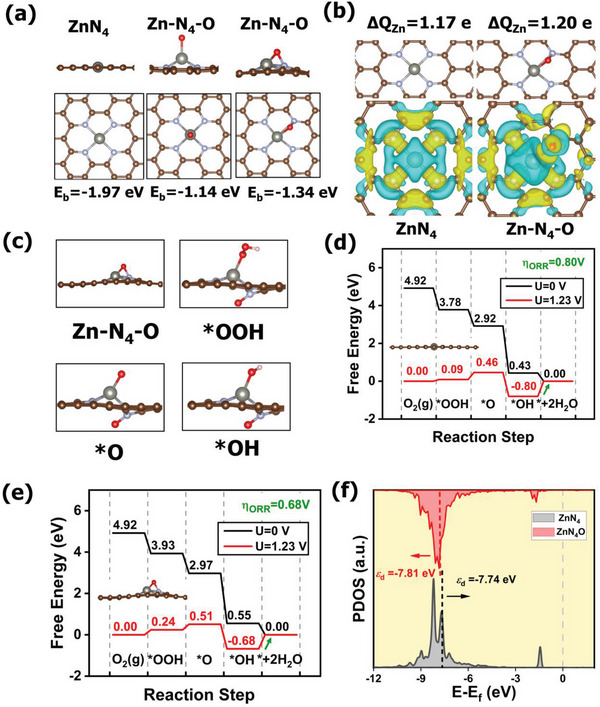
a) Different coordination environments of Zn single atoms and corresponding calculated binding energy. b) Bader charge on the Zn center, geometric model of Zn single atoms, and charge difference density, where the charge accumulation and depletion are illustrated by yellow and cyan, respectively. c) Zn‐N_4_‐O model and adsorbed reaction intermediates (*OOH, *O, and *OH). Free energy diagram for ORR on d) Zn‐N_4_ and e) Zn‐N_4_‐O at the equilibrium potentials of U = 0 V and U = 1.23 V. f) PDOS curves of Zn‐N_4_‐O and Zn‐N_4_.

The influence of axial O on the local coordination geometry and electron configuration of Zn centers was initially investigated based on density functional theory (DFT) calculations. As shown in **Figure** **1**a, one Zn‐N_4_ model and two possible Zn‐N_4_‐O models were considered. In two Zn‐N_4_‐O models, the O atom possesses different coordination environments: one is an O atom solely bonded with the Zn atom, and the other one is an O atom at the bridge site bonded with both N and Zn atoms. To determine the relative stability of the above three models, we calculated their binding energies (*E*_b_). The results show that the *E*_b_ value of −1.97e V for Zn‐N_4_ is the lowest, which means that Zn atom coordinated with four in‐plane nitrogen atoms is more energetically favored than that with two five‐fold coordination. Furthermore, we find that O atom prefer to adsorb at the bridge site between N and Zn atoms (*E*_b_ = −1.34 eV) rather than solely bonded with Zn atom (*E*_b_ = −1.14 eV). Therefore, the model of Zn‐N_4_‐O as shown in the right panel of Figure 1a is considered for further calculations. The schematic diagram in Figure 1b presents the models of Zn‐N_4_ and Zn‐N_4_‐O. It is clearly seen that the characteristic plane Zn‐N_4_ configuration is distorted upon the introduction of O atom at the bridge site accompanied with variation of the electronic properties. The electronic localization functions (ELF) illustrate that Zn‐N_4_ shows a symmetric electron structure (Figure S1a, Supporting Information). On the contrary, significant charge polarization and distorted electronic symmetry are observed in Zn‐N_4_‐O caused by the traction of O coordination (Figure S1b, Supporting Information). Meanwhile, the incorporation of O atom induces charge transfer in the Zn center of Zn‐N_4_‐O (ΔQ_Zn_ = 1.20 e) more than that of Zn‐N_4_ (ΔQ_Zn_ = 1.17 e) (Figure 1b), indicating higher oxidation state of Zn ions when five‐fold being coordinated. This ligand effect is also be reflected by the charge difference, where the distribution of the charge density in Zn‐N_4_‐O is more asymmetrical than that in Zn‐N_4_, consistent with the structural features of them.

Next, the modulation of the ORR activity was investigated. The fully relaxed configurations of the substrate of Zn‐N_4_‐O and adsorbed reaction intermediates (*OOH, *O, and *OH) of ORR are shown in Figure 1c. Note that the adsorption of the reaction intermediates induces the structural evolution of Zn‐N_4_‐O to Zn‐N_3_‐O under ORR working conditions. Nevertheless, after desorption of the OH species from the site of Zn‐N_3_‐O, the Zn‐N_4_‐O will then be recovered and involved into the next cycle of ORR process. As illustrated by the free energy changes (Figure 1d,e), all steps of the elementary reaction steps for Zn‐N_4_‐O and Zn‐N_4_ are exothermic processes at *U* = 0 V. The free energy changes at *U* = 1.23 V reveals that the last electron transferred step of the desorption of *OH with the highest energy barrier is the rate determining step (RDS) for both Zn‐N_4_ and Zn‐N_4_‐O. Therefore, the overpotential of ORR over Zn‐N_4_ moiety is calculated to be 0.8 V, whereas Zn‐N_4_‐O active center gives rise to a lower overpotential of 0.68 V. It means that Zn‐N_4_‐O possesses superior ORR activity to Zn‐N_4_, which is derived from the optimized adsorption‐desorption behaviors of oxygen intermediates on active site in Zn‐N_4_‐O. In other words, the excessive adsorption of *OH on Zn‐N_4_ leads to its relatively poor catalytic performance for ORR. In comparison, upon the deposition of O ligand on Zn‐N_4_, the adsorption free energy of *OH shifts to a higher energy, demonstrating the more effortless desorption step on Zn‐N_4_‐O site.

For in‐depth insight into origin of activity enhancement mechanism for Zn‐N_4_‐O, the *d*‐electron orbital modulation of the Zn center induced by axial O coordination was further investigated. The *d*‐band center theory is an effective tool for predicting the interaction strength of surface and adsorbates. As shown in Figure 1f, the *d*‐band center of Zn site in Zn‐N_4_‐O (−7.81 eV) is lower than that in Zn‐N_4_ (−7.74 eV). The downshifted *d*‐band center suggests the antibonding orbital is filled with more electrons, resulting in the weakened binding energies for *OH on the Zn‐N_4_‐O.^[^ ^28^, ^29^ ^]^ The electronic interaction between the Zn center and the *OH intermediate was also explored by the variation of Zn‐3*d* orbitals before and after adsorbing *OH (Figure S2, Supporting Information). The *d*‐band centers of Zn atoms upshift 2.41 and 2.76 eV in Zn‐N_4_‐O and Zn‐N_4_ after *OH adsorption, respectively. Compared with Zn‐N_4_, the smaller change in *d*‐band center of Zn‐N_4_‐O demonstrates the less electrons in the *d* orbitals involved in stabilizing *OH, thereby weakening the adsorption strength of *OH. In general, the introduction of axial O coordination induces the electron transfer from Zn center to neighboring atoms and achieves electronic delocalization, which can optimize the adsorption strength of *OH and lower the energy barrier of RDS, resulting in enhanced ORR performance.

### 2.2 Synthesis and Characterizations of the Zn‐N_4_‐O and Zn‐N_4_

**Figure 2:**
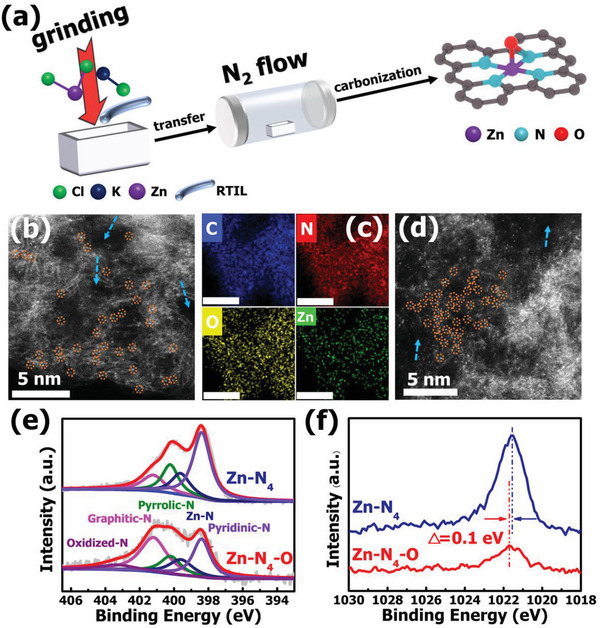
a) A schematic illustration for the synthesis route of Zn‐N_4_‐O. b) Aberration‐corrected high‐angle annular dark‐field scanning TEM image of Zn‐N_4_‐O. c) Elemental mapping image of Zn‐N_4_‐O (scale bar, 20 nm). d) Aberration‐corrected high‐angle annular dark‐field scanning TEM images of Zn‐N_4_. e) N 1s spectra of Zn‐N_4_ and Zn‐N_4_‐O. f) Zn 2p spectra of Zn‐N_4_ and Zn‐N_4_‐O.

Inspired by the impressive modulating effect of axial O on central Zn site and optimized ORR kinetic process by theoretical predictions, here we developed an ionic liquid‐assisted molten salt template strategy to synthesize the asymmetric Zn‐N_4_‐O SACs, as shown in **Figure** **2**a. The molten salts template is a powerful route for preparation of porous carbon structure.^[^ ^30^ ^]^ Eutectic salt mixtures of ZnCl_2_ and KCl are used as solid‐phase template, in which ZnCl_2_ is also used as the Zn source to form the single atomic structure. The room‐temperature ionic liquid (RTIL) as a low melting points organic salt exhibits many useful advantages, including durable thermal and chemical natures, high ionic conductivity, and particularly tunable composition.^[^ ^31^, ^32^, ^33^ ^]^ Here, the RTIL 1‐ethyl‐3‐methylimidazolium dicyanamide is served as solvents to dissolve ZnCl_2_ at room temperature and as a carbon source, providing sufficient N sites to capture the Zn atoms during carbonization step. The oxygen‐containing impurities could be trapped by KCl solid matrix and occupy the halide sites of KCl crystal, forming stabilized O^−^ ion in the ground solid‐phase templates.^[^ ^34^, ^35^ ^]^ Thus, it is anticipated to change the coordination environment of Zn single atom by controlling the carbonization temperature of the RTIL‐assisted molten salts, and prepare a unique Zn atomic coordination to break the symmetry of the electronic structure. Then, the samples are prepared by finely controlling carbonization temperature of 800, 850, 900, and 950 °C. The X‐ray diffraction (XRD) patterns of samples suggest two diffraction peaks of graphitic carbon at 26° and 43°, and no characteristic peaks related to Zn compounds, implying the atomic dispersion of Zn species in all prepared samples (Figure S3, Supporting Information). High resolution transmission electron microscopy (HR‐TEM) image exhibits that all samples possess similar morphology of carbon structure, consisting of irregular hollow carbon layer structures. Neither Zn clusters nor particles can be observed in the carbon structure (Figure S4a–d, Supporting Information). To clearly elucidate evolution of carbon structure at various annealing temperatures, we further performed the Raman measurement. As shown in Figure S5a–d (Supporting Information), the Raman spectra can be fitted by four different deconvoluted peaks. Two major peaks are located at ≈1343 cm^−1^ (D_1_) and ≈1597 cm^−1^(G), which can be assigned to defect/disordered structure of graphene with A_1g_ vibration and sp^2^‐graphitic structure with E_2g_ vibration, respectively. The D_4_ and D_3_ peaks at ≈1200 and 1500 cm^−1^ corresponds to amorphous structure in carbon and a disordered graphitic lattice/polyene‐like structures.^[^ ^36^ ^]^ Among all samples, the sample annealed at 950 °C shows the lowest value of 1.12, indicating the highest graphitization degree of carbon support. Moreover, the graphitic structure size is identified by ratio of integrated intensity of D_1_ peak and G peak (Equation 7 in Experiment Section). With increasing temperature, the average graphene domain size L_a_ increases from ≈9.2 to ≈10 nm. Hence, the 950 °C sample holds the largest graphitic structure and better conductivity, which is favorable to the electron mobility during the catalytic process. Meanwhile, the decrease in the *I*_D4_/*I*_G_ and *I*_D3+D4_/*I*_G_ ratios of high‐temperature annealed samples demonstrates that there is a tendency to form defects under low temperature conditions, and these defects (such as amorphous carbon) can be diminished by increasing the carbonization temperature.^[^ ^37^ ^]^ The pure carbon sample N‐C without addition of ZnCl_2_ was also prepared through a similar synthetic route (see Supporting Information). Figure S5e (Supporting Information) presents that the *I*_D1_/*I*_G_ ratio of 950 °C sample is lower than that of pure carbon N‐C sample, which is derived from the formation of disordered sites on carbon matrix by introduction of Zn atoms.

To intuitively illustrate microenvironment of the Zn single atom catalysts, the samples annealed at 950 °C (named as Zn‐N_4_‐O) and 800 °C (named as Zn‐N_4_) will be the focus of discussion in the following. Aberration‐corrected high‐angle annular dark‐field scanning TEM (AC HAADF‐STEM) images of Zn‐N_4_‐O obviously reveals that the isolated Zn atom are homogenously distributed in the porous carbon structure (atomic Zn sites marked by orange circles and pores on carbon marked by red arrows) (Figure 2b; Figure S6, Supporting Information). The elemental mapping demonstrates uniform distribution of C, N, O and Zn in the carbon matrix of Zn‐N_4_‐O (Figure 2c). It is noting that the density of Zn single atoms in Zn‐N_4_‐O is obviously reduced compared with Zn‐N_4_ (Figure 2d; Figure S7, Supporting Information). Due to the volatility feature of Zn species, Zn atoms are inevitably evaporated from the carbon matrix at higher temperature, resulting in the lower density of Zn single atoms in Zn‐N_4_‐O. Even so, partial Zn atoms are still preserved and the contents of Zn in Zn‐N_4_‐O and Zn‐N_4_ determined by ICP‐AES are 0.43 wt.% and 2.50 wt.%, respectively. (Table S1, Supporting Information). Figure S8 (Supporting Information) clearly shows the presence of abundant pores in the carbon supporting of both Zn‐N_4_‐O and Zn‐N_4_, and the porous nature of carbon structure is further demonstrated by Brunauer‐Emmett‐Teller (BET) analysis. The Zn‐N_4_‐O and Zn‐N_4_ present significant specific surface areas, which are 1862 and 2030 m^2^ g^−1^, respectively (Figure S9, Supporting Information). The X‐ray photoelectron spectroscopy (XPS) was employed to analyze the chemical states of samples. Figure S10 (Supporting Information) indicates the existence of C, N, O and Zn species in samples annealed at different temperatures. As N and Zn species evaporate at higher temperatures, the atom ratio of Zn species of 0.78% in Zn‐N_4_ (800 °C) reduced to 0.22% in Zn‐N_4_‐O (950 °C), while the atom ratio of N species decreases from 12.5% to 3.48%. As exhibited in Figure 2e, the N 1s XPS spectrum of Zn‐N_4_‐O can be deconvoluted into five peaks, corresponding to graphitic‐N (401.2 eV), pyrrolic‐N (400.2 eV), Zn‐N bonds (399.6 eV), pyridinic‐N (398.4 eV) and oxidized‐N (403‐405 eV).^[^ ^19^, ^38^ ^]^ Figure S11 (Supporting Information) shows the absences of Zn‐N bonds in pure carbon N‐C sample, proving the Zn atoms in Zn‐N_4_‐O are captured by carbon supporting and retained during the pyrolysis process. This result is consistent with AC HAADF‐STEM image observation. Figure 2f presents that the Zn 2p_3/2_ peaks for Zn‐N_4_‐O and Zn‐N_4_ is located at 1021.70 and 1201.55 eV, respectively, demonstrating the electronic states of Zn in Zn‐N_4_‐O and Zn‐N_4_ is close to +2.^[^ ^14^ ^]^ Notably, The Zn 2p_3/2_ peak for Zn‐N_4_‐O is located at a slightly higher bonding energy than Zn‐N_4_ counterpart. This variation in electronic state of Zn species suggests that Zn‐N_4_‐O possibly possesses a different coordination environment on central Zn. Meanwhile, in O 1s spectrum for samples can be divided into four peaks of C‐O, C = O, O‐H and oxygen with low coordination (Figure S12, Supporting Information).^[^ ^39^, ^40^ ^]^ The O 1s XPS spectrum presents that the intense peak for low coordination oxygen can be clearly observed in Zn‐N_4_ and that significantly decreases in Zn‐N_4_‐O. Considering the peak of Zn 2p_3/2_ in Zn‐N_4_‐O shifts to a higher energy compared with Zn‐N_4_, the result may indicate that the Zn site is served as an electron donor as well as transfers its electrons to unsaturated coordinated O site.

**Figure 3:**
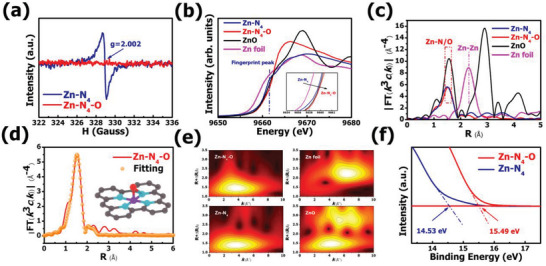
a) ESR spectra for Zn‐N_4_ and Zn‐N_4_‐O. b) Zn K‐edge XANES spectra and c) k^3^‐weighted Fourier transform of EXAFS spectra of Zn‐N_4_, Zn‐N_4_‐O, ZnO and Zn foil. d) EXAFS fitting curves of Zn‐N4‐O in R space. e) Wavelet transform k3‐weighted EXAFS spectra of Zn‐N_4_, Zn‐N_4_‐O, ZnO and Zn foil. f) UPS spectra of Zn‐N_4_ and Zn‐N_4_‐O.

The electron spin resonance (ESR) is a powerful approach to the issues of electronic structure and is further used to disclose the unpaired electrons in samples. The g‐factor of 2.002 for Zn‐N_4_ is attributed to the unpaired electron of oxygen sites in Zn‐N_4_ sample and the signal of g‐factor is silent in Zn‐N_4_‐O sample, affirming the presence of saturated coordination oxygen in Zn‐N_4_‐O (**Figure** **3**a).^[^ ^41^, ^42^, ^43^ ^]^ In addition, this ESR signal is also detected in the corresponding pure carbon sample (the same annealing temperature as Zn‐N_4_) (Figure S13, Supporting Information). The detailed coordination environment and chemical state for atomically dispersed Zn species were analyzed by Zn K‐edge X‐ray absorption spectra (XAS). Figure 3b shows the absorption edge of Zn‐N_4_ is located between Zn foil and ZnO, indicating the valence state of Zn in Zn‐N_4_ is between 0 and +2. The Zn‐N_4_‐O exhibits a similar edge position to ZnO, demonstrating the valence state of atomic Zn is close to +2 and the higher energy of the edge position is attributed to the introduction of Zn‐O coordination. The prominent peak of white‐line for Zn‐N_4_‐O is higher than that for Zn‐N_4_, confirming the higher oxidized electronic state of central Zn atom in Zn‐N_4_‐O.^[^ ^44^ ^]^ These results are well in consist with the XPS analysis. Meanwhile, the difference in the positions of white‐line between Zn‐N_4_‐O and Zn‐N_4_ also manifests the change in the configuration environment of Zn site. Moreover, the fingerprint peak for typical D_4h_ symmetry of Zn phthalocyanine at 9661.8 eV can be observed in Zn‐N_4_, suggesting that the Zn center of Zn‐N_4_ is a planar structure. However, this fingerprint peak is absent in Zn‐N_4_‐O, which indicates that the geometric configuration of the Zn site changes from a planar structure in Zn‐N_4_ to a non‐planar structure by additional binding of axial coordination.^[^ ^45^, ^46^ ^]^ The extended X‐ray adsorption fine structure (EXAFS) displays that there is no main peaks of the Zn‐Zn path in Zn‐N_4_‐O and Zn‐N_4_, verifying the atomic dispersions of Zn sites (Figure 3c). The main peaks for Zn‐N_4_‐O and Zn‐N_4_ located at 1.53 and 1.49 Å are attributed to the backscattering Zn and light atoms (N, O). The peak for the Zn‐N_4_‐O shifts toward a longer value of R due to the presence of axial ligand and the similar phenomenon also can be observed in previous works.^[^ ^47^, ^48^ ^]^ The geometry structure of Zn atoms in the Zn‐N_4_‐O and Zn‐N_4_ are acquired by EXAFS fitting (Figure 3d; Figures S14 and S15, Supporting Information). The results illustrate that the Zn‐N_4_‐O fitted well with the model that the Zn atom coordinated with four in‐plane nitrogen atoms and one axial direction O atom. The best fitting result of Zn‐N_4_ sample is Zn atom coordinated with in‐plane four nitrogen atom. The corresponding structural parameters extracted from the EXAFS fitting are exhibited in Table S2 (Supporting Information). The optimized fitting results present the coordination number (CN) of 4.8 for Zn‐N_4_‐O with the average bond length of 2.03 Å and the CN of 4.3 for Zn‐N_4_ with the average bond of 1.97 Å. The larger coordination number value strongly suggests that the Zn‐O bond is formed on the axial direction of ZnN_4_ structure. Besides, the EXAFS wavelet transform (WT) analysis was performed due to its high resolution in both K‐ and R‐spaces (Figure 3e). The WT contour maximum for Zn‐N_4_‐O and Zn‐N_4_ are located at k = 3.7 and 3.6 Å^−1^, verifying the coordination environment change between Zn‐N_4_‐O and Zn‐N_4_,^[^ ^27^ ^]^ and the WT contour maximum at 6.4 Å^−1^ for Zn‐Zn bond is not observed in both Zn‐N_4_‐O and Zn‐N_4_, confirming the atomically dispersed Zn atoms. Hence, the obtained results revealed Zn site in Zn‐N_4_‐O sample is coordinated with four nitrogen atoms and one axial O atom, and the Zn site is coordinated with in‐plane four nitrogen atoms in Zn‐N_4_ sample. Given this, the effect of axial O ligand on the electron structure of Zn‐N_4_‐O was investigated by ultraviolet photoemission spectroscopy (UPS). The cut off energy (E_cutoff_) of Zn‐N_4_‐O and Zn‐N_4_ is 15.49 and 14.53 eV, respectively (Figure 3f). Based on the equation: E_Φ_ = hv‐**|**E_cutoff_‐E_F_ **|**, the work function of Zn‐N_4_‐O and Zn‐N_4_ are identified as 5.71 and 6.67 eV, respectively. The lower value of work function of Zn‐N_4_‐O renders that the active electron density of Zn‐N_4_‐O is effectively regulated.^[^ ^26^, ^49^ ^]^ The UPS result from an experimental point of view to verify the DFT calculations. Combined with the results of Bader charge (Figure 1b), the relatively reduced the electrons on Zn center weaken the binding strength with oxygen‐containing intermediates, reducing the blockage of intermediates on the Zn‐N_4_‐O surface.

### 2.3 Electrocatalytic Properties

**Figure 4:**
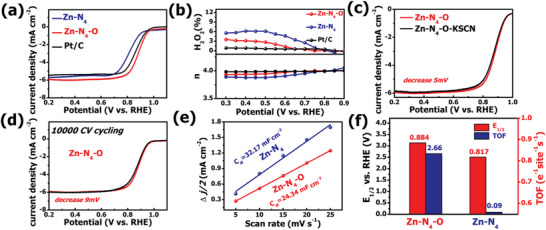
a) ORR polarization curves of Zn‐N_4_, Zn‐N_4_‐O and Pt/C in O_2_‐saturated 0.1 m KOH solution. b) H_2_O_2_ yield and electron transfer number of Zn‐N_4_‐O, Zn‐N_4_ and Pt/C from 0.3 to 0.9 V. c) ORR polarization curves of Zn‐N_4_‐O before and after addition of 0.01 KSCN in 0.1 m KOH. d) Polarization curves of Zn‐N_4_‐O before and after 10 000 cycles. e) The double‐layer charging current density difference plotted as a function of scan rate fitted to a linear regression. f) Calculated TOF values of Zn‐N_4_‐O and Zn‐N_4_.

Since the Zn‐N_4_‐O holds the unusual structure and enhanced activity predicted by theoretical calculations, thus we further investigated the ORR electrocatalytic activity of Zn‐N_4_‐O using three‐electrode construction in O_2_‐saturated 0.1 m KOH electrolyte. The LSV analysis exhibits that Zn‐N_4_‐O displays a favorable ORR activity with a half‐wave potential (E_1/2_) of 0.884 V, which is close to commercial Pt/C (0.881 V) and significantly higher than Zn‐N_4_ (0.817 V) (**Figure** **4**a). Besides, the N‐C sample without incorporation of Zn single atoms shows the negligible ORR activity (Figure S16, Supporting Information). As predicted by the theoretical calculations, the experimental results present the superior catalytic activity of Zn‐N_4_‐O structure, confirming that regulation of the electronic state of center Zn by O coordination is in favor of boosting ORR process. The catalytic activities of Zn‐N_4_‐O can compete with those of other earth‐abundant ORR electrocatalysts (Table S3, Supporting Information). The polarization curves at various rotating speeds were performed to calculate the number of transferred electrons. According to the Koutechy‐Levich (K‐L) equation, the average number is ≈4 (Figure S17, Supporting Information). Meanwhile, the reaction pathway of Zn‐N_4_‐O was investigated by the rotating ring disk electrode (RRDE) measurement (Figure 4b). The value of electron transfer number is in range from 3.93–4.00 and the H_2_O_2_ yield in 0.1 m KOH electrolyte is less than 4% in the reduction potential range from 0.3–0.9 V, confirming a direct four electron pathway during ORR process on the Zn‐N_4_‐O. A preferred 4‐electron pathway with low H_2_O_2_ yield effectively suppresses the degeneration on activity caused by Fenton reaction and mitigates the corrosion of the carbon supporting. The poison experiments were performed to explore the influence of axial O coordination on affinity of center metal to SCN^−^ ion. As shown in Figure 4c and Figure S18 (Supporting Information) the half‐wave potential of Zn‐N_4_ structure decrease ≈17 mV after addition 0.01 m KSCN, while the poisoning Zn‐N_4_‐O only displays ≈5 mV of degenerated half‐wave potential, presenting the enhanced tolerance of Zn‐N_4_‐O to the SCN^−^ ions. Without the axial coordination on active central metal site, the SCN^−^ ion would bind to metal site through S atom, resulting in the formation of axial *SCN adsorbate. Due to the high negative adsorption energy of *SCN adsorbate at metal center, the active sites would be blocked, thus leading to the decay on the ORR activity.^[^ ^50^ ^]^ The positive effect of axial coordination is also observed in analogous electrocatalysts.^[^ ^51^ ^]^ Li and co‐workers have shown that the existence of axial hydroxyl ligand is able to vary the adsorption free energy of *SCN, weakening the poison effect of SCN^−^ on central Fe site. In combination with above discussion, the result led us to conclude that the improved tolerance to the SCN^−^ ions of Zn‐N_4_‐O can be attributed to oxygen bonded with the Zn site in axial direction, which hinders the central Zn from being poisoned by adsorption of SCN^−^ ion. Meanwhile, Zn‐N_4_‐O shows favorable tolerance ability to the methanol (Figure S19, Supporting Information). Only slight decay on current is observed for Zn‐N_4_‐O after injecting methanol, while the Pt/C present an obvious current drop. The long‐term stability was measured using galvanostatic procedure at 0.5 V versus RHE with a rotating speed of 1600 rpm and accelerated durability test (ADT) by 10 000 CVs (Figure 4d; Figure S20, Supporting Information). Impressively, the current density provided by Zn‐N_4_‐O stays 90.1% after 35 h of continuous operation and the LSV curves show a negligible degradation on half‐wave potential (9 mV) after 10 000 CVs, indicating the extremely high durability of Zn‐N_4_‐O. To further illustrate the structural stability of Zn single atom, we characterized the Zn‐N_4_‐O catalysts after ADT. As shown in HR‐TEM image of post‐ORR Zn‐N_4_‐O (Figure S21, Supporting Information), no presence of Zn clusters nor particles is observed in the carbon structure, suggesting that the Zn single atoms do not aggregate during the ORR process. The AC HAADF‐STEM images confirm that the Zn species are still atomically dispersed on the carbon architecture after catalytic process (Figure S22a–c, Supporting Information). The elemental mapping demonstrates uniform distribution of C, N, O and Zn in the carbon matrix of post‐ORR Zn‐N_4_‐O (Figure S22d, Supporting Information). Furthermore, Zn 2p XPS spectrum shows that the intensity of Zn peak slightly decreases after ADT, which suggests partial Zn single atoms leach out during the catalytic process (Figure S23a, Supporting Information). The N 1s XPS spectrum presents that the Zn‐N bond is remained in the post‐ORR Zn‐N_4_‐O (Figure S23b, Supporting Information). The above characterization results indicate that the Zn‐N_4_‐O holds a decent structural stability during the ORR process. Figure S24 (Supporting Information) exhibits a small Tafel slope of Zn‐N_4_‐O (83 mV dec ^−1^), lower than that of Zn‐N_4_ (128 mV dec^−1^), demonstrating a faster ORR kinetics of the Zn‐N_4_‐O. It is well established that the catalytic activity of a catalyst is positively dependent on the intrinsic nature of per isolated active site and density of active sites. Thus, the electrochemical double‐layer capacitance (C_dl_) was calculated based on the CV curves at different scanning rate in non‐faradic region to determine the electrochemically active surface area (ECSA), which is regarded as an indicator for available active surface area of the catalysts (Figure 4e; Figure S25, Supporting Information). The value of C_dl_ and ECSA for Zn‐N_4_‐O are estimated to be 24.34 mF cm^−2^ and 119.5 cm^2^_ECSA_ on the working electrode with the measured area of 0.19625 cm^2^, less than those for Zn‐N_4_ (32.17 mF cm^−2^ of C_dl_ and 157.8 cm^2^_ECSA_ of ECSA). Then the normalized activity by ECSA was employed to illustrate the intrinsic activity of Zn‐N_4_‐O and Zn‐N_4_ sites. Clearly, although the smaller available electrochemically active surface area in Zn‐N_4_‐O catalyst, which is derived from the volatility feature of Zn species at high pyrolysis temperature, the Zn‐N_4_‐O presents the more favorable ORR performance than Zn‐N_4_, suggesting a higher intrinsic ORR activity per active site in Zn‐N_4_‐O (Figure S26, Supporting Information). To illustrate this point, the TOF was calculated to exhibit the difference more intuitively between the intrinsic activity of Zn‐N_4_‐O and Zn‐N_4_. At 0.85 V versus RHE, the Zn‐N_4_‐O shows a much higher TOF of 2.66 e^−1^ site^−1^ s^−1^ than that for Zn‐N_4_ (0.09 e^−1^ site^−1^ s^−1^) (Figure 4f). To explore the active sites in Zn‐N_4_‐O sample, we further employed more comprehensive poison experiments (Figures S27 and S28, details see caption of Figure S28, Supporting Information). Additionally, Zn‐N_4_‐O also exhibits superior ORR activity to Zn‐N_4_ in acid electrolyte and the half‐wave potential of Zn‐N_4_‐O is 0.742 V (Figure S29, Supporting Information). The above experimental results are consistent with the theoretical prediction that the introduction of axial O coordination on Zn site can indeed promote intrinsic catalytic activity in ORR process.

### 2.4 Zn‐Air Battery Measurement

**Figure 5:**
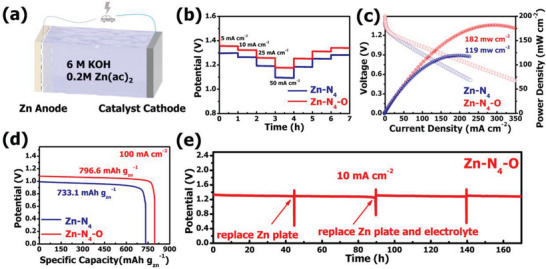
a) Schematic of the assembled zinc‐air battery. b) Galvanostatic discharge of Zn‐N_4_ ZAB and Zn‐N_4_‐O ZAB under various current density. c) Polarization and corresponding power density curves for Zn‐N_4_ ZAB and Zn‐N_4_‐O ZAB. d) discharge curves of Zn‐N_4_ ZAB and Zn‐N_4_‐O ZAB at a current density of 100 mA cm^−2^. e) Long‐term durability measurement of Zn–air battery assembled with Zn‐N4‐O cathode at 10 mA cm^−2^.

Considering the effective catalytic activity of Zn‐N_4_‐O catalyst, a Zn‐Air battery (ZAB) was assembled to present its potential application. The ZAB was conducted by utilizing Zn‐N_4_‐O and Zn‐N_4_ as cathode electrocatalysts and a Zn plate as anode (**Figure** **5**a). The ladder‐shaped discharge measurement at various current densities (from 5 to 50 mA cm^−2^) is shown in Figure 5b. Obviously, the Zn‐N_4_‐O ZAB can offer a higher discharge voltage than Zn‐N_4_ ZAB at each constant current density, especially at large current density. The Zn‐N_4_‐O ZAB displays a flat discharging plateau of ≈1.18 V at 50 mA cm^−2^ current densities and its discharging plateau can recover reversibly when the current density returns to 5 mA cm^−2^. The results demonstrate the decent rate performance and reversibility of Zn‐N_4_‐O ZAB. The ZAB assembled by Zn‐N_4_‐O can provide a peak power density of 182 mW cm^−2^, which is much larger than that of Zn‐N_4_ ZAB (119 mW cm^−2^) (Figure 5c). Figure 5d presents the specific capacity of ZABs normalized to the consumption of Zn. Under a large operation current density of 100 mA cm^−2^, the specific capacity and energy density of the battery conducted with Zn‐N_4_‐O reaches 796.6 mAh g^−1^ and 828.3 Wh g^−1^, while those of Zn‐N_4_ battery are only 733.1 mAh g^−1^ and 690.6 Wh kg^−1^ (Figure S30, Supporting Information). The galvanostatic discharge test was conducted at 10 mA cm^−2^ to further investigate durability of assembled Zn‐N_4_‐O ZAB. As shown in Figure 5e, no obvious degeneration on discharge voltage plateau can be observed before the zinc anode is exhausted, indicating the robust stability of Zn‐N_4_‐O cathode. The battery can operate continuously for least 160 h by replacing the zinc plates. Notably, the voltage can return to the original state after replenishing the zinc plate and electrolyte, which further validates the ultra‐stable feature of Zn‐N_4_‐O.

## 3 Conclusion

In conclusion, an axial ligand O regulation strategy is brought to break the symmetry of geometry/electronic structure in Zn‐N_4_ moiety, realizing obviously enhanced ORR activity. DFT calculations reveal that the introduction of axial O coordination is capable of evoking electronic delocalization central Zn and further reduces the electrons participated in stabilizing *OH, resulting in optimized adsorption‐desorption behaviors of oxygen intermediates on Zn‐N_4_‐O sites. As a result, the Zn‐N_4_‐O presents high intrinsic ORR activity with superior turn‐over frequency, which is orders of magnitude higher than that of Zn‐N_4_. Impressively, the ZAB constructed by Zn‐N_4_‐O can deliver the peak power density of 182 mW cm^−2^ and a robust long‐term durability for over 160 h. This work not only opens a new route for regulating the microenvironment of single atom catalysts but also offers a new opportunity for designing and developing effective electrocatalysts served in energy conversion and storage devices.

## Conflict of Interest

The authors declare no conflict of interest.

## Supporting information

Supporting Information

## Data Availability

The data that support the findings of this study are available from the corresponding author upon reasonable request.
