# Supplementary material for: Axial Oxygen Ligands Regulating Electronic and Geometric Structure of Zn‐N‐C Sites to Boost Oxygen Reduction Reaction
Source: Adv Sci (Weinh). 2023 Jun 26;10(24):2302152. doi: 10.1002/advs.202302152 (PMC10460851; doi:10.1002/advs.202302152)
Supplement: Supplementary file 1 — Supporting Information [file ADVS-10-2302152-s001.pdf]

ADVANCED SCIENCE
Open Access

Supporting Information

for *Adv. Sci.*, DOI 10.1002/advs.202302152

Axial Oxygen Ligands Regulating Electronic and Geometric Structure of Zn-N-C Sites to Boost Oxygen Reduction Reaction

Qiuyan Jin, Chenhui Wang, Yingying Guo, Yuhang Xiao, Xiaohong Tan, Jianpo Chen, Weidong He, Yan Li, Hao Cui* and Chengxin Wang**

Supporting Information

Axial oxygen ligands regulating electronic and geometric structure of Zn-N-C sites to boost oxygen reduction reaction

*Qiuyan Jin, Chenhui Wang, Yingying Guo, Yuhang Xiao, Xiaohong Tan, Jianpo Chen, Weidong He, Yan Li *, Hao Cui *, Chengxin Wang **

State Key Laboratory of Optoelectronic Materials and Technologies, School of Materials Science and Engineering, Sun Yat-sen University, Guangzhou 510275, China.

The Key Laboratory of Low-Carbon Chemistry & Energy Conservation of Guangdong Province, Sun Yat-sen University, Guangzhou 510275, China

Corresponding Author Email Address:

E-mail:cuihao3@mail.sysu.edu.cn (H. Cui), liyan266@mail.sysu.edu.cn (Y. Li), wchengx@mail.sysu.edu.cn (C. Wang)

Keywords: Single atom catalyst • Axial ligands regulation • Zn-based material • Oxygen reduction reaction • Zinc-air battery

Experimental sections

2.1 Chemicals.

Potassium hydroxide (KOH), perchloric acid ($HClO_4$), sulfuric acid (H_2SO_4, 98%) and potassium chloride (KCl) were purchased from Aladdin, Pt/C (20% wt) and Zinc chloride ($ZnCl_2$, 98%) was obtained from Alfa Aesar. 1-ethyl-3-methylimidazolium dicyanamide ($C_9H_{12}N_4$) was obtained from Zhengzhou Acme Chemical Co. LTD. All reagents were used as delivered without further purification

2.2 Preparation of the Zn-N_4-O and Zn-N_4 structure.

Typically, 2g $ZnCl_2$ was ground with 1 mL 1-ethyl-3-methylimidazolium dicyanamide to form a uniform white slurry. Subsequently, 1g KCl was mixed with the slurry and further ground. The obtained mixture was transferred to a porcelain boat, followed by heating process at 300 ℃ for 1h and then carbonization at 950 ℃ for 1h under N_2 atmosphere (heating rate of 10 ℃ min^{-1}). The as-synthesized material was washed with 0.5 M H_2SO_4, a great deal of water and ethanol. Finally, the black powdery product was dried at 80 ℃ overnight and ground at room temperature, defined as Zn-N_4-O. The Zn-N_4 powder was prepared following the similarly procedure expect that the carbonization temperature was at 800 ℃.

2.3 Characterizations of Zn-N_4-O and Zn-N_4 structure.

The microstructure and morphologies of samples were observed by TEM (FEI TALOS F200X) operating at 200 kV. The D-MAX 2200 VPC Rigaku X-ray diffraction (XRD) patterns with Cu Kα radiation (40 kV, 26 mA) was employed to character crystal structure. Renishaw inVia was taken using a 532 nm excitation laser to collect the Raman spectra. Chemical composition were obtained on the ESCALAB250 XPS measurements equipped with Al Kα (1486.6 eV) X-ray source. The electron spin resonance (ESR) spectrum were measured on JES-FA200 ESR spectrometer system. Adsorption data of samples was used for the Brunauer-Emmett-Teller (BET, ASAP2460) surface area calculations. The Barrett-

Joyner-Halenda (BJH) method was used to calculate the pore size distributions. The inductively coupled plasma atomic emission spectrometer (ICP-AES) was carried out on an Agilent 5110 (Agilent Technologies). Ultraviolet photoemission spectroscopy (UPS) experiments were measured on an PHI5000 Versaprobe III (PHI Instruments). Zn *K*-edge analysis was performed with Si(111) crystal monochromators at the BL11B beamlines at the Shanghai Synchrotron Radiation Facility (SSRF) (Shanghai, China). Before the analysis at the beamline, samples were pressed into thin sheets with 1 cm in diameter and sealed using Kapton tape film. The XAFS spectra were recorded at room temperature using a 4-channel Silicon Drift Detector (SDD) Bruker 5040. Zn K-edge extended X-ray absorption fine structure (EXAFS) spectra were recorded in fluorescence excitation mode. Negligible changes in the line-shape and peak position of Zn K-edge XANES spectra were observed between two scans taken for a specific sample. The spectra were processed and analyzed by the software codes Athena and Artemis.

2.4 Electrochemical Measurements.

The ORR performance of samples was tested in three-electrode cell using Bio-logic VMP3 workstation equipped with a Pine Modulated Speed Rotator (Pine Research Instrumentation, USA). The glassy carbon, a graphite electrode and a Ag/AgCl electrode with saturated KCl were employed as working electrode, counter electrode and reference electrode, respectively. The testing electrolyte was 0.1 M KOH or 0.1 M $HClO_4$. The fabrication of working electrode: 5 mg powdery catalyst was dispersed in a mixed solution, which contains 20 μL Nafion ionomer solution (5 wt %) and 980 μL absolute ethanol. The above solution was further sonicated for 2h to form a homogeneous catalyst ink. Subsequently, 16 μL of as-prepared catalyst ink was loaded for measurements. The mass loading of catalyst was approximate 0.4 mg cm^{-2}. For comparison, 16 μL of Pt/C (20 wt %) ink was dropped on the electrode for ORR performance test. The cyclic voltammetry (CV) experiments collected at a scan rate of 50 mV s^{-1}. The ORR polarization curve measurements were performed at various

rotating speeds from 400 to 2000 rpm. According to the previous work, note that the positive scan was recorded for the Pt/C catalyst to remove the hysteresis effect and the negative scan was used for all Zn-N-C electrocatalysts.

The Koutecky-Levich (K-L) equation was used to defined electron transfer number (n) and kinetic current density (J_k).

$$\frac{1}{J} = \frac{1}{J_L} + \frac{1}{J_K} = \frac{1}{B\omega^{0.5}} + \frac{1}{J_K} \quad (1)$$

$$B = 0.62\, nFC_0 (D_0)^{2/3} v^{-1/6} \quad (2)$$

$$J_K = nFkC_0 \quad (3)$$

Where J, J_L and J_k are measured, diffusion- and kinetic-limiting current densities, respectively. ω represents the electrode ratation rate. B is determined by slope of the Koutechy-Levich (K-L) plot based on Levich equation. F is the Faraday constant (F = 96485 C mol^{-1}), D_0 (1.93 × 10^{-5} cm^2 s^{-1}) and C_0 (1.26 × 10^{-6} mol cm^{-3}) are the diffusion and concentration coefficient of O_2. v (0.01009 cm^2 s^{-1}) is the kinetic viscosity.

The peroxide percentage and the electron transfer number (n) were calculated with the following equations:

$$HO_2^- \% = 200 \times \frac{I_R / N}{I_D + I_R / N} \quad (4)$$

$$n = 4 \times \frac{I_D}{I_D + I_R / N} \quad (5)$$

I_D and I_R are disk and ring current. N is determined to be 0.37, which is current collection efficiency. The electrochemically active surface area (ECSA) was defined by measuring capacitive current associated with double-layer charging versus the scan rating (C_{dl}). The ECSA of a sample is calculated from the C_{dl} according to the equation: ECSA = S*C_{dl}/C_s, where the C_s is the specific capacitance of the catalyst. A general specific capacitance of Cs = 0.040 mF cm^{-2} is accepted based on typical reported value [30]. S is the actual area of the working electrode. Turnover frequency (TOF) of catalytically active Zn-N4-O and Zn-N4

sites can be obtained according to the equation:

$$TOF = \frac{J_k \times N_e}{\omega_{metal} \times C_{Cat} \times N_A \;/\; M_{metal}} \qquad (6)$$

where J_k is the kinetic current density, N_e (6.24×10^{18}) is the electron number per Coulomb. ω_{metal} is the Zn content in catalysts. The ICP-AES suggest the Zn contents for Zn-N_4 and Zn-N_4-O are 2.50 wt % and 0.43 wt %. C_{cat} is the catalyst loading, N_A is the Avogadro constant (6.022×10^{23}), and M_{metal} is the mass per mole of metal.

The average graphene domain size (L_a) was calculated with the following equations:

$$La = (2.4 \times 10^{-10}) \times \lambda_l^4 \times \left(\frac{A_{D1}}{A_G}\right)^{-1} \qquad (7)$$

Where λ_l is the excitation laser wave length, A_{D1} and A_G represent the integrated intensity of D1 and G peaks. L_a is an average graphene domain size.

2.5 Zn-Air Battery measurement.

5 mg catalyst powder was dispersed in a mixed solution containing 20 μL Nafion (5 wt %) and 980 μL absolute ethanol. Then, the mixture was further sonicated for 2h to form a homogeneous catalyst ink. The catalyst ink was loaded on the carbon paper (1*1 cm^{-2}) for Zn-Air measurement. The mass loading of catalyst was approximate 1 mg cm^{-2}. The Zn plate and carbon paper were employed as anode and cathode assembled into a home-made Zn-air battery with 6 M KOH and 0.2 M $Zn(Ac)_2$ aqueous solutions served as electrolyte. Zn-air batteries were evaluated under ambient conditions.

Computational details

We performed the density functional theory (DFT) based calculations [1] by using the Vienna Ab initio Simulation Package (VASP) [2, 3], which enabled us to achieve the relaxed geometries and total energies. We adopted the projector augmented wave (PAW) [4, 5] method describe the nuclei–electron interactions. The Perdew–Burke–Ernzerhof (PBE) [6, 7] functional within the generalized gradient approximation (GGA) [8, 9] was employed to calculate the

exchange−correlation energy. The van der Waals interactions were included using the Grimm's D3 method [10]. The kinetic cutoff energy was set to 520 eV. A vacuum layer of 20 Å was employed in order to cancel the interaction of adjacent layers along the z-direction. We constructed the model of the single Zn atom supported by the N doped graphene using an orthorhombic cell with *a* = 12.824, b = 12.339 and c = 20.000 Å, containing 54 C atoms, 4 N atoms and 1 Zn atom (Figure 1a). Γ-centered *k*-mesh grids [11] were set to be 2 × 2 × 1, 3 × 3 × 1 and 7 × 7 × 1 for structural relaxation, self-consistent field and density of states (DOS) calculations, respectively. The full convergence of the structural relaxations were obtained when the force on each atom was less than 0.02 eV/Å. The solvation effect was considered and over dielectric constant (εr) is set to 80. DFT+U calculations with Hubbard U = 4.12 eV for Zn was carried out in order to explore the catalytic properties of the single Zn atoms supported by the N doped graphene [12] .

The average energy of *d* band near the Fermi energy for transition metal atoms is calculated by

$$d_{center} = \frac{\int_{-\infty}^{+\infty}(\varepsilon\times\rho)d\varepsilon}{\int_{-\infty}^{+\infty}\rho d\varepsilon} \qquad \text{Eq.1}$$

where ρ is the electron density at certain energy level of ε. The binding energy (E_b) is calculated by

$$E_b = E_{tot} - E_{sub} - E_{atom} \qquad \text{Eq. 2}$$

where E_{tot} – E_{sub} – E_{atom} represent the energies of ZnN4/ZnN4-O, energy of the substrate without Zn atom and single Zn atom, respectively.

Based on the works of Nøskov et al.,[13] the following equations were used to illustrate the elementary steps of ORR and OER. The ORR proceeds via the following steps.

$$O_2\,(g) + H_2O + e^- + {}^* \rightarrow {}^*OOH + OH^- \qquad \text{Eq.3}$$

$$^*OOH + e^- \rightarrow {}^*O + OH^- \qquad \text{Eq.4}$$

$$^*O + H_2O + e^- \rightarrow {}^*OH + OH^- \qquad \text{Eq.5}$$

$^{*}OH + e^{-} \rightarrow OH^{-} + {}^{*}$ Eq.6

In the above equations, * refers to the substrate and (g) stands for the gas phase. The Gibbs free energy change (ΔG) was calculated for each electron-transfer step, which is equal to the free energy difference between the initial and final states according to[14]:

$\Delta G = \Delta E - T\Delta S + \Delta ZPE$ Eq.7

where ΔE refers to the energy differences between product and reactant states achieved by DFT calculations, ΔS is the entropy difference and ΔZPE stands for the change of zero-point energy.

We calculated the adsorption free energy ΔG_{*OOH}, ΔG_{*O}, ΔG_{*OH} and ΔG_{*H} based on the following equations:

$\Delta G_{*OOH} = G_{*OOH} - G_{*} - (2G_{H2O} - 3/2G_{H2})$ Eq.8

$\Delta G_{*O} = G_{*O} - G_{*} - (G_{H2O} - G_{H2})$ Eq.9

$\Delta G_{*OH} = G_{*OH} - G_{*} - (G_{H2O} - 1/2G_{H2})$ Eq.10

$\Delta G_{*H} = G_{*H} - G_{*} - 1/2G_{H2}$ Eq.11

Therefore, the reaction free energy of Eqs. 2 – 5 (ΔG_1, ΔG_2, ΔG_3, ΔG_4) for ORR can be obtained based on the equations:

$\Delta G_1 = \Delta G_{*OOH} - 4.92$ Eq.12

$\Delta G_2 = \Delta G_{*O} - \Delta G_{*OOH}$ Eq.13

$\Delta G_3 = \Delta G_{*OH} - \Delta G_{*O}$ Eq.14

$\Delta G_4 = -\Delta G_{*OH}$ Eq.15

Then we calculated the overpotential (η^{ORR}) for ORR based on the equation:

$\eta^{ORR} = 1.23 + \max(\Delta G_{1,} \Delta G_{2,} \Delta G_{3,} \Delta G_4)$ Eq.16

Reference

[1] W. Kohn, L.J. Sham, Self-Consistent Equations Including Exchange and Correlation Effects, Phys. Rev., 140 (1965) A1133-A1138.

[2] J. Hafner, Ab-initio simulations of materials using VASP: Density-functional theory and beyond, J. Comput. Chem., 29 (2008) 2044-2078.

[3] G. Kresse, J. Furthmüller, Efficient iterative schemes for ab initio total-energy calculations using a plane-wave basis set, Phys. Rev. B, 54 (1996) 11169-11186.

[4] P.E. Blöchl, O. Jepsen, O.K. Andersen, Improved tetrahedron method for Brillouin-zone integrations, Phys. Rev. B, 49 (1994) 16223-16233.

[5] P.E. Blöchl, Projector augmented-wave method, Physical Review B, 50 (1994) 17953-17979.

[6] J.P. Perdew, Y. Wang, Accurate and simple analytic representation of the electron-gas correlation energy, Phys. Rev. B, 45 (1992) 13244-13249.

[7] J.P. Perdew, J.A. Chevary, S.H. Vosko, K.A. Jackson, M.R. Pederson, D.J. Singh, C. Fiolhais, Atoms, molecules, solids, and surfaces: Applications of the generalized gradient approximation for exchange and correlation, Phys. Rev. B, 46 (1992) 6671-6687.

[8] J.P. Perdew, K. Burke, M. Ernzerhof, Generalized Gradient Approximation Made Simple, Phys. Rev. Lett., 77 (1996) 3865-3868.

[9] G. Kresse, D. Joubert, From ultrasoft pseudopotentials to the projector augmented-wave method, Physical Review B, 59 (1999) 1758-1775.

[10] D.G.A. Smith, L.A. Burns, K. Patkowski, C.D. Sherrill, Revised Damping Parameters for the D3 Dispersion Correction to Density Functional Theory, The Journal of Physical Chemistry Letters, 7 (2016) 2197-2203.

[11] H.J. Monkhorst, J.D. Pack, Special points for Brillouin-zone integrations, Phys. Rev. B, 13 (1976) 5188-5192.

[12] P. Sun, Z. Qiao, S. Wang, D. Li, X. Liu, Q. Zhang, L. Zheng, Z. Zhuang, D. Cao, Atomically Dispersed Zn-Pyrrolic-N4 Cathode Catalysts for Hydrogen Fuel Cells, n/a e202216041.

[13] J.K. Nørskov, J. Rossmeisl, A. Logadottir, L. Lindqvist, J.R. Kitchin, T. Bligaard, H. Jónsson, Origin of the Overpotential for Oxygen Reduction at a Fuel-Cell Cathode, J. Phys. Chem. B, 108 (2004) 17886.

[14] H.A. Hansen, J. Rossmeisl, J.K. Nørskov, Surface Pourbaix diagrams and oxygen reduction activity of Pt, Ag and Ni(111) surfaces studied by DFT, Physical Chemistry Chemical Physics, 10 (2008) 3722-3730.

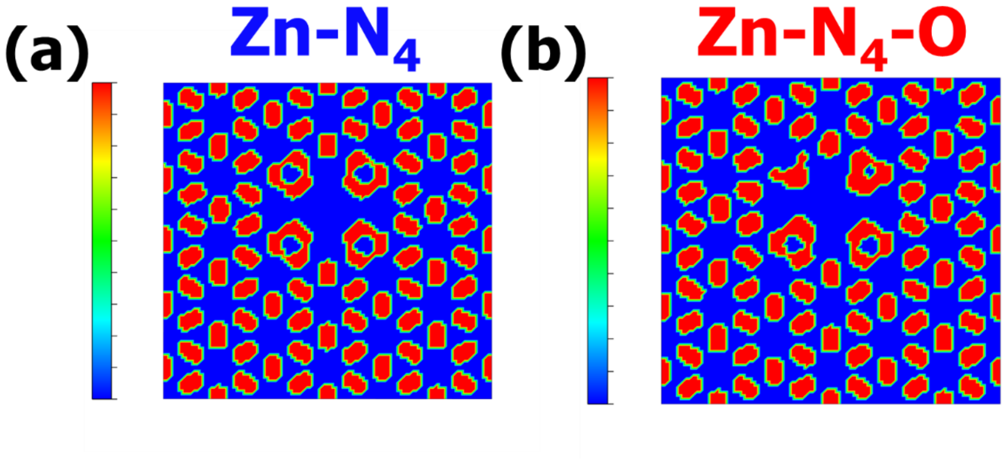

Figure S1. Electron localization functions for (a) Zn-N_4 and (b) Zn-N_4-O model.

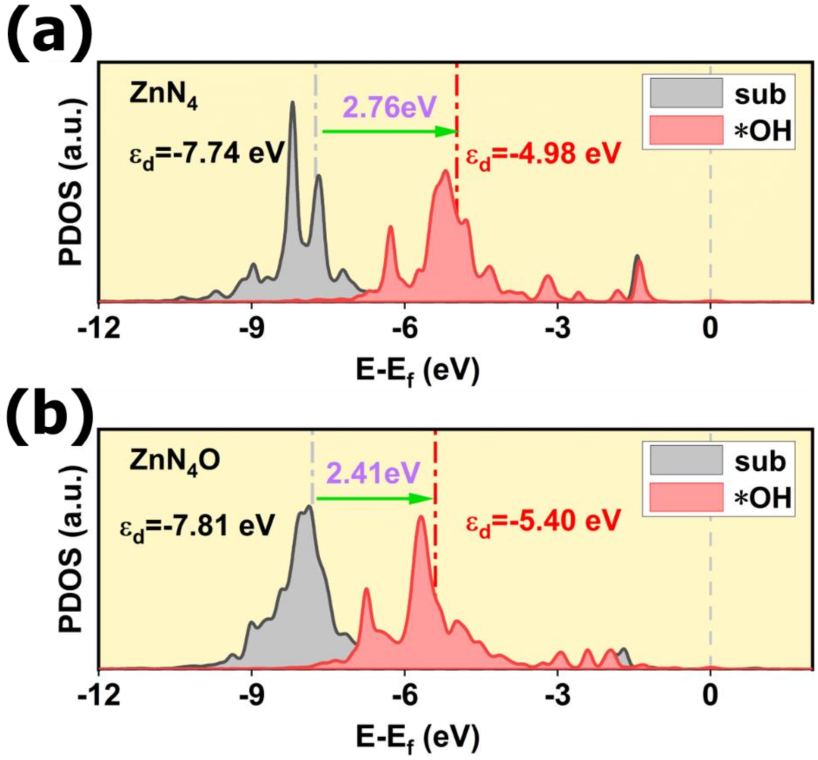

Figure S2. DOS of the (a) Zn-N_4 and (b) Zn-N_4-O models before and after *OH interaction.

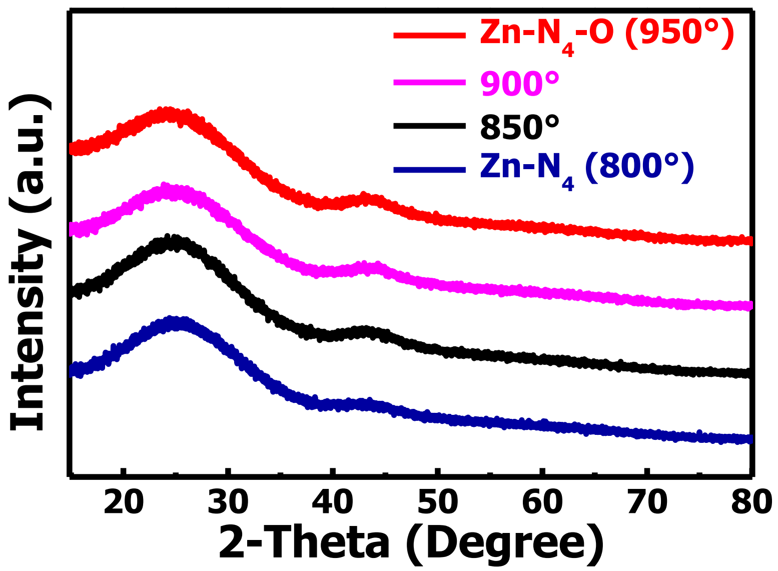

Figure S3. XRD pattern of sample annealed at 800 °C, 850 °C, 900 °C and 950 °C.

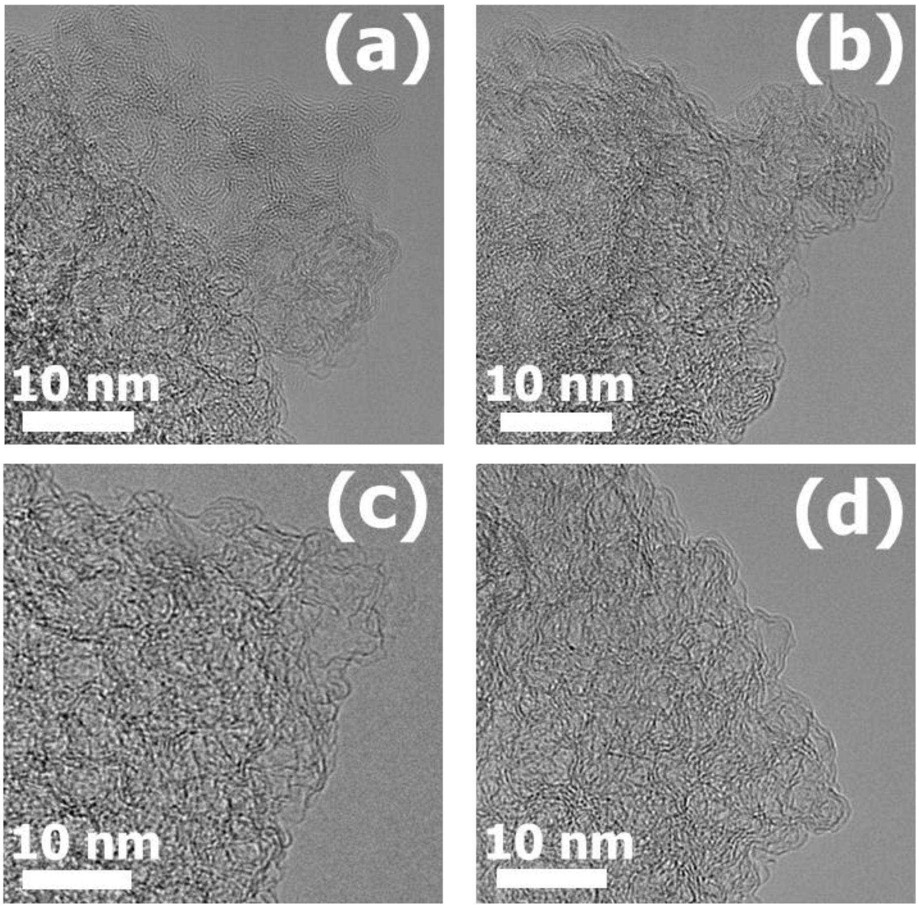

Figure S4. HR-TEM images of samples annealed at (a) 800 ℃, (b) 850 ℃, (c) 900 ℃, and (d) 950 ℃.

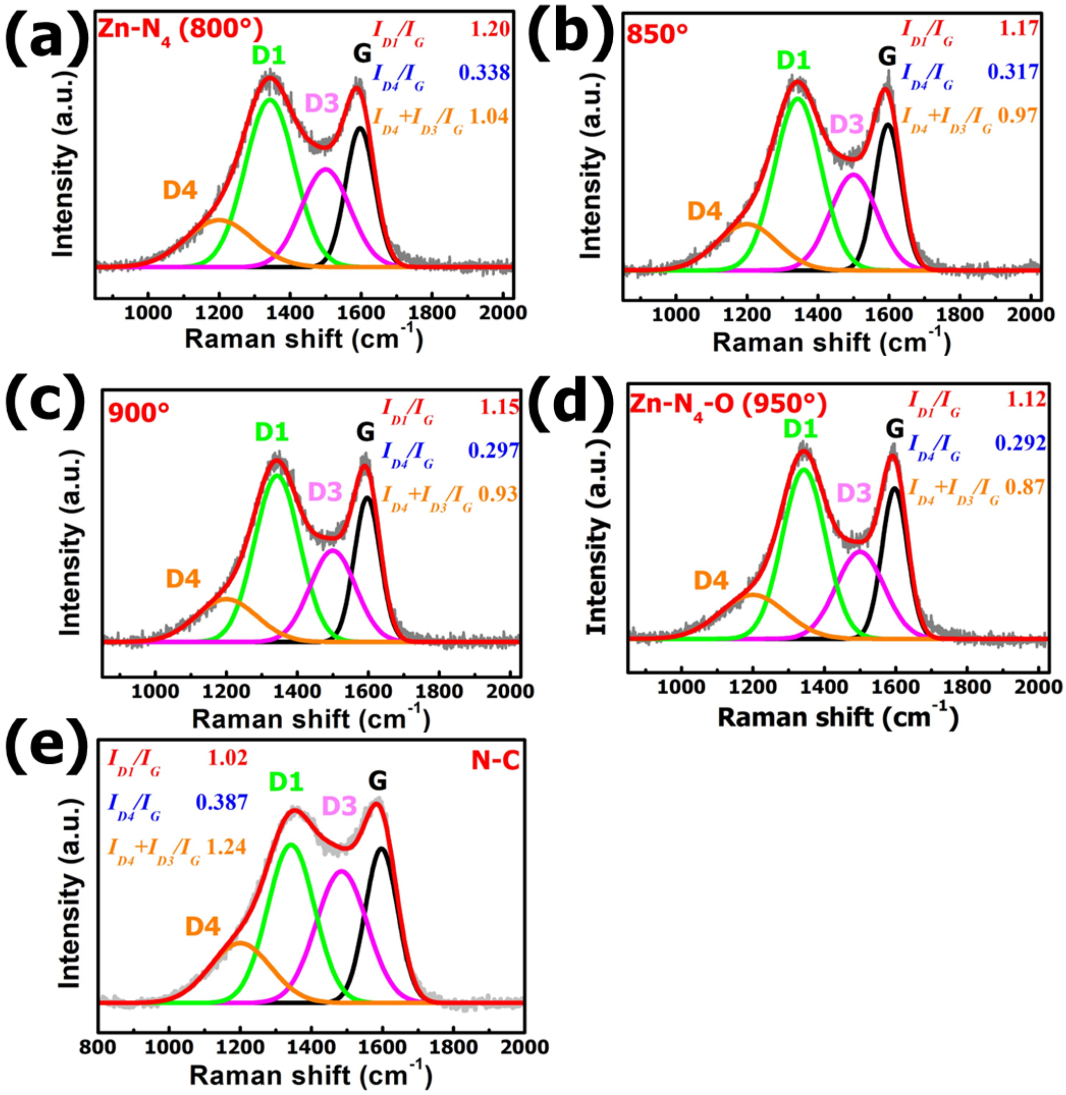

Figure S5. Raman spectra of samples annealed at (a) 800 ℃, (b) 850 ℃, (c) 900 ℃, (d) 950 ℃ and (e) Raman spectra of pure carbon sample.

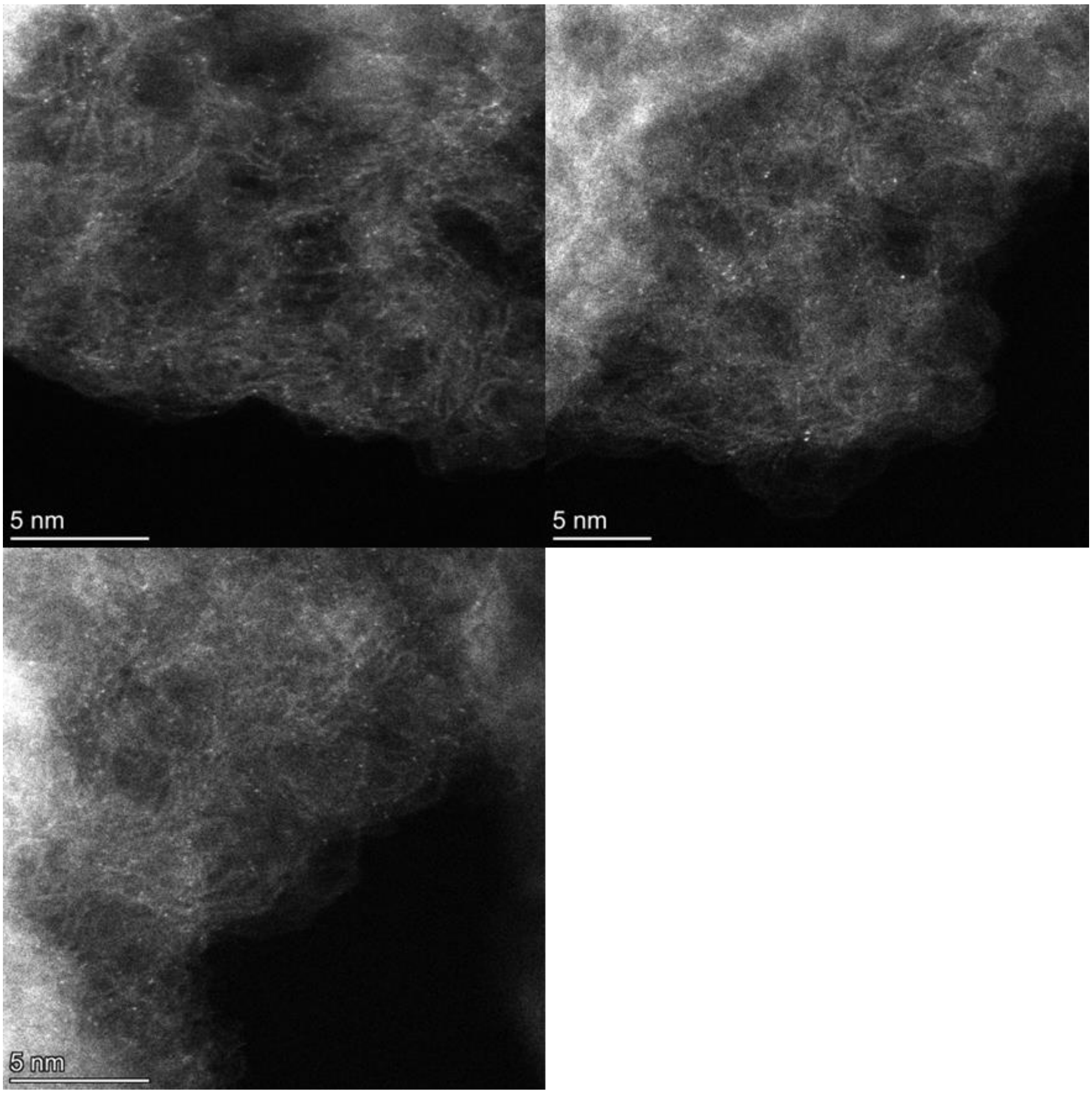

Figure S6. Aberration-corrected high-angle annular dark-field scanning TEM images of Zn-N_4-O.

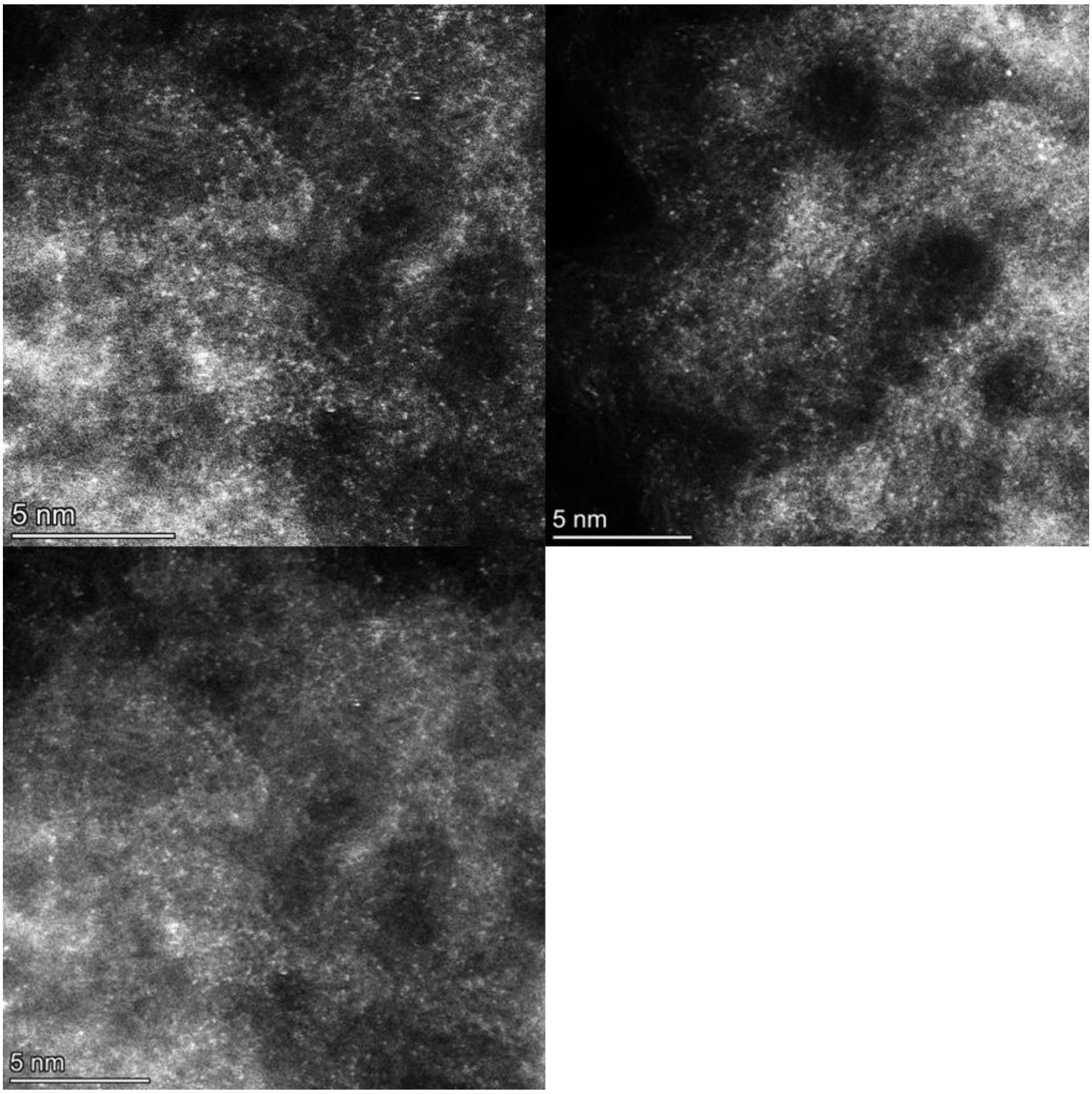

Figure S7. Aberration-corrected high-angle annular dark-field scanning TEM images of Zn-N_4.

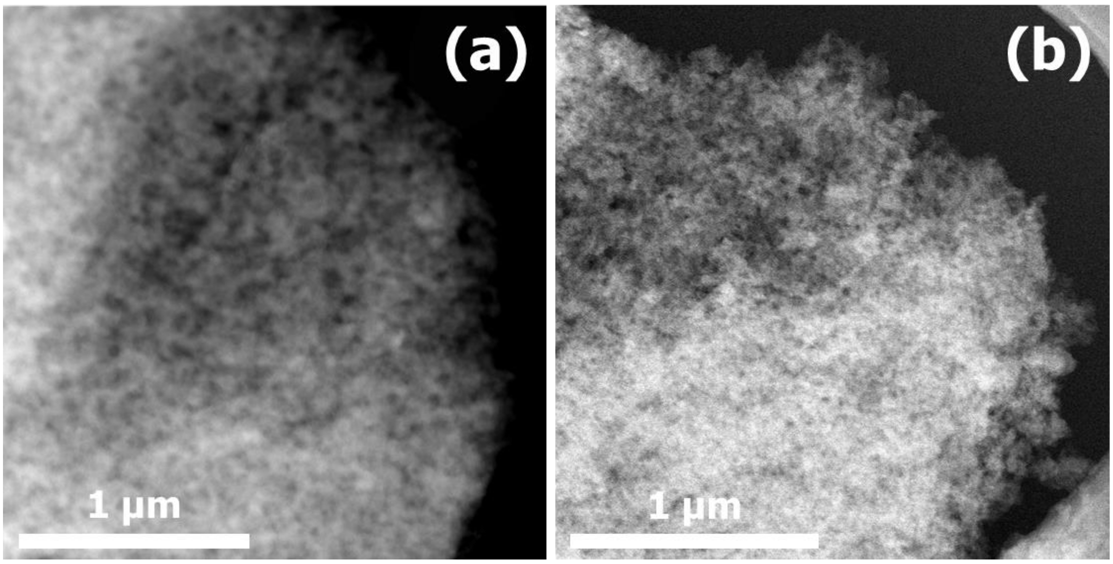

Figure S8. HAADF-STEM image of (a) Zn-N_4-O and (b) Zn-N_4.

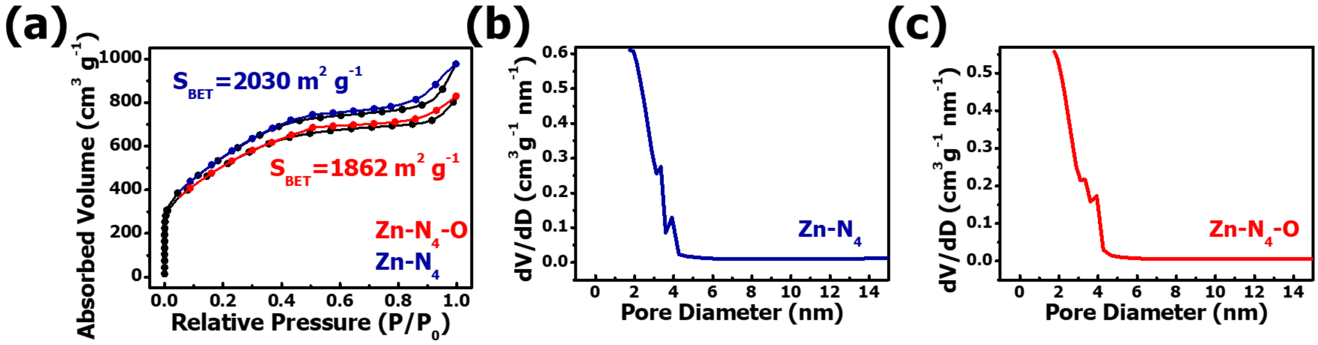

Figure S9. (a) N_2 absorption-desorption isotherms of Zn-N_4-O and Zn-N_4. Pore size distribution curves of (b) Zn-N_4 and (c) Zn-N_4-O.

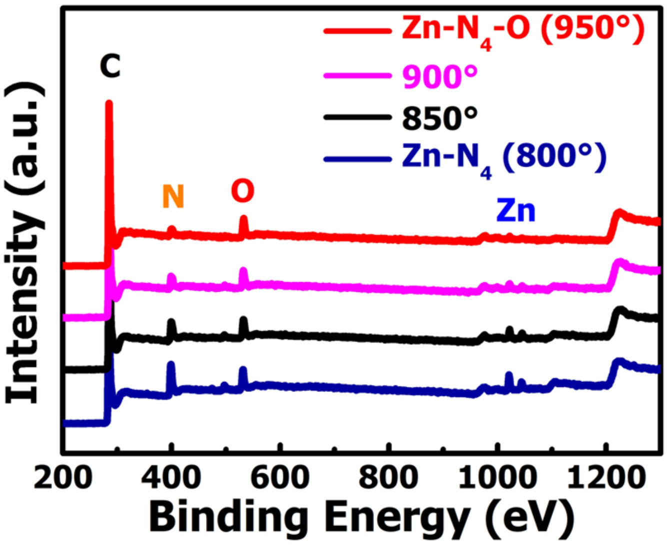

Figure S10. XPS survey spectra of sample annealed at 800 ℃, 850 ℃, 900 ℃ and 950 ℃.

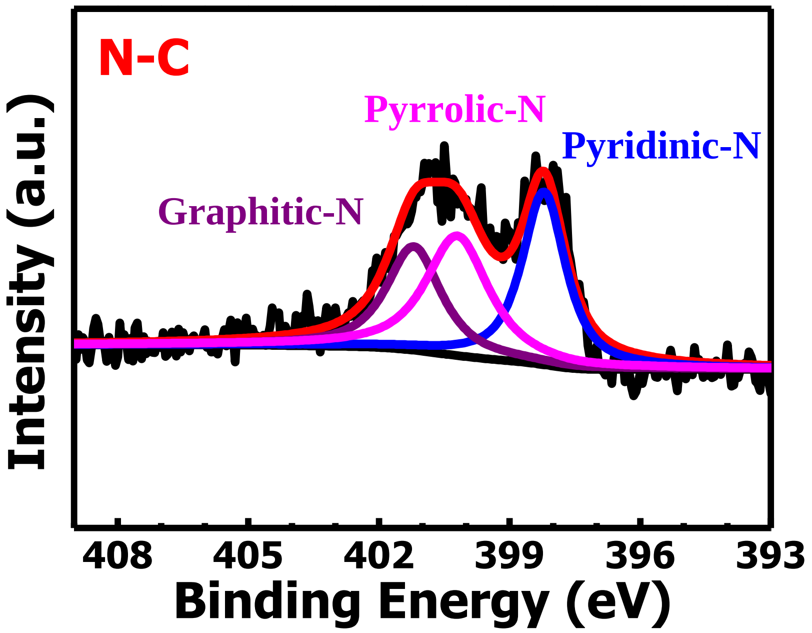

Figure S11. N 1s XPS spectra of N-C.

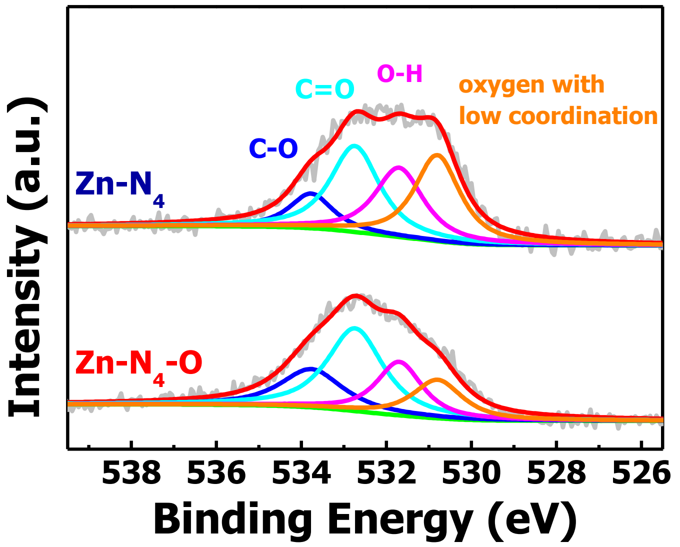

Figure S12. O 1s XPS spectra of Zn-N_4-O and Zn-N_4.

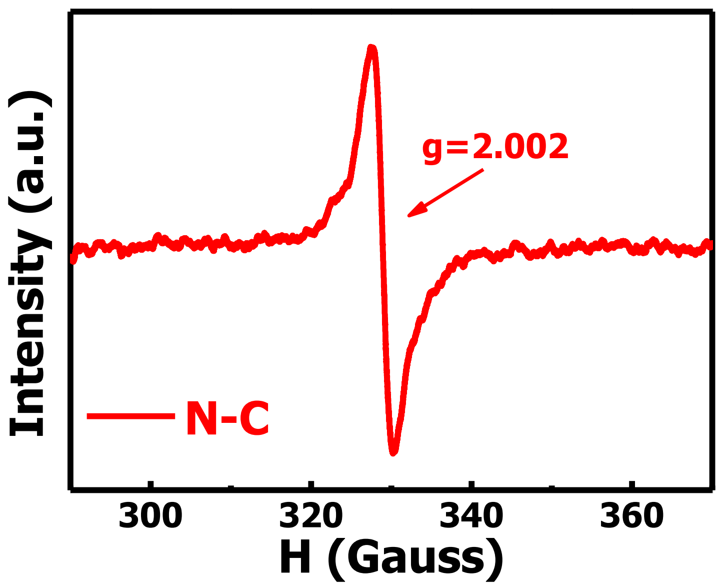

Figure 13. ESR spectra for corresponding pure carbon.

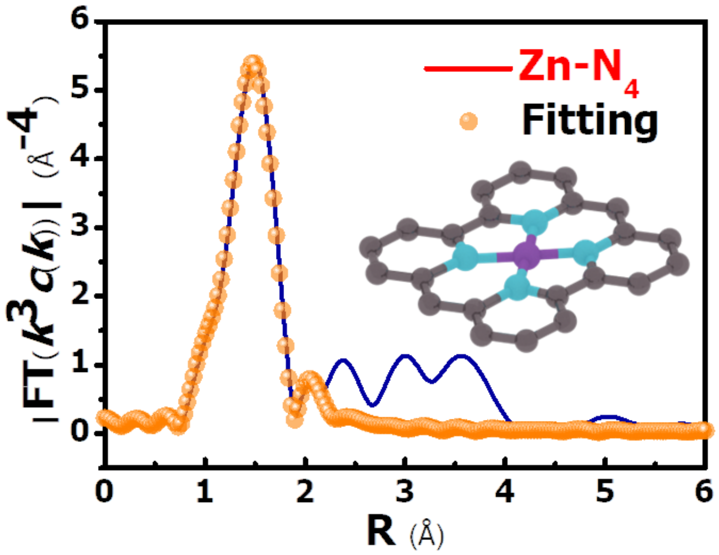

Figure S14. The fitted curve and experimental data of Zn-N_4 in R-space.

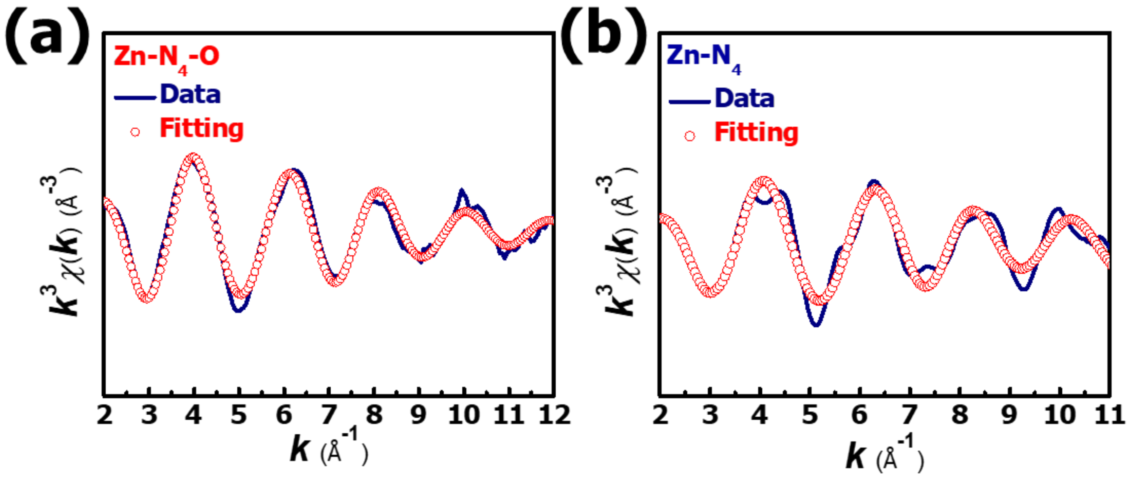

Figure S15. The fitted curve and experimental data of (a) Zn-N_4-O and (b) Zn-N_4 in k-space.

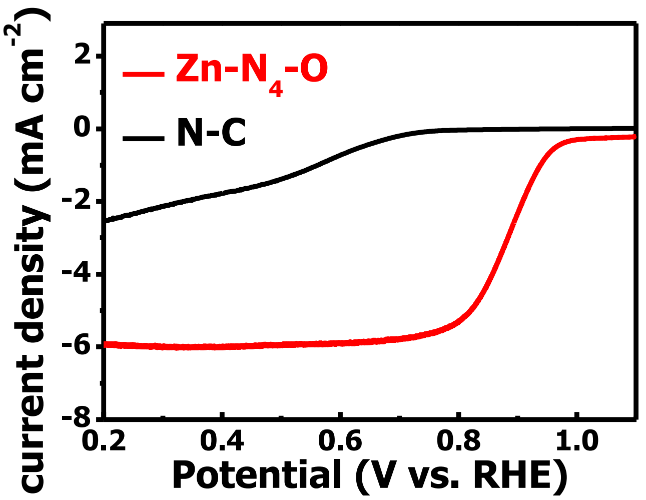

Figure S16. ORR polarization curves of Zn-N_4-O and N-C in 0.1 M KOH.

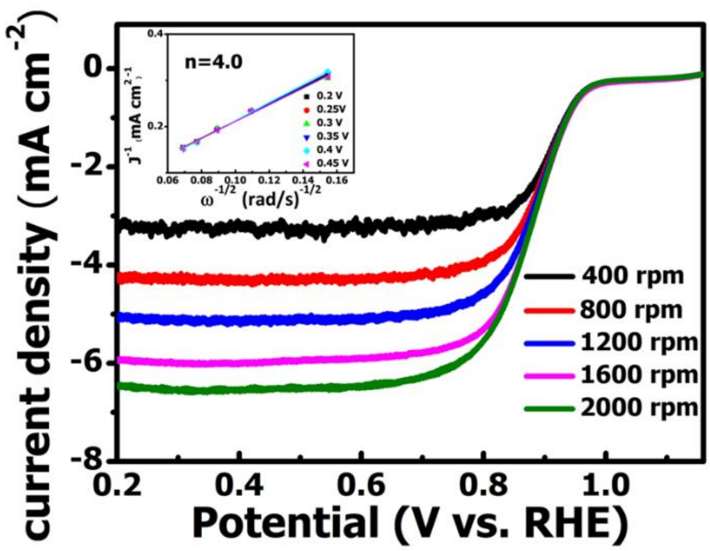

Figure S17. Polarization curves of Zn-N_4-O with different roatating speeds.

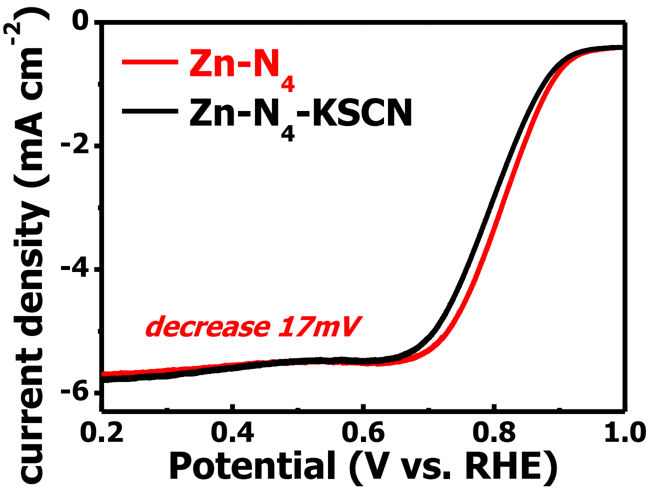

Figure S18. ORR polarization curves of Zn-N_4 before and after addition of 0.01 KSCN in 0.1 M KOH.

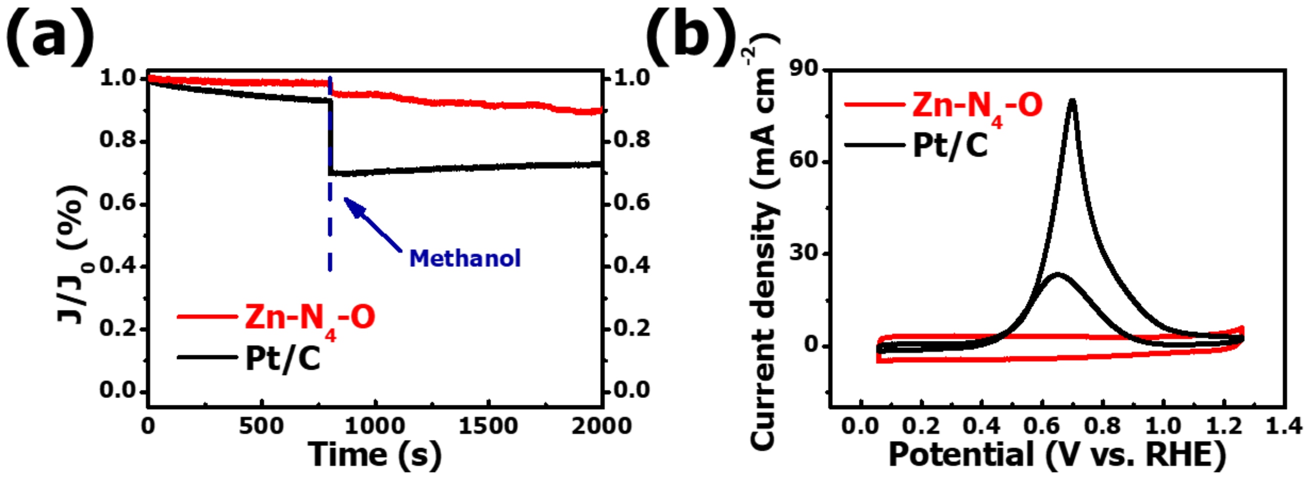

Figure S19. (a) chronoamperometric curves of a methanol crossover test with Zn-N_4-O and Pt/C. (b) CVs of Pt/C and Zn-N_4-O in 1 M KOH with 1.0 M CH_3OH at a scan rate of 50 mV/s.

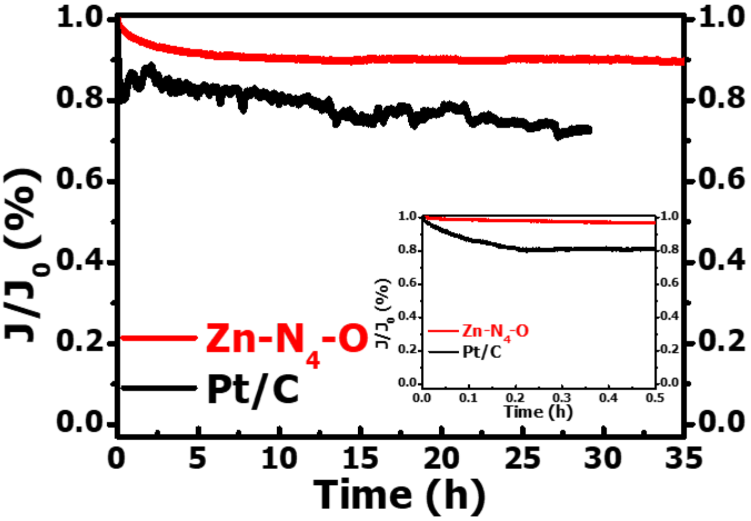

Figure S20. The stability test for Zn-N_4-O and Pt/C with a rotation speed of 1,600 RPM in O_2-saturated 0.1 M KOH solution.

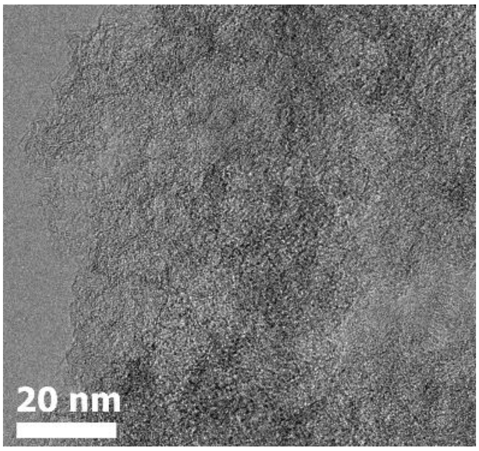

Figure S21. HR-TEM image of post-ORR Zn-N_4-O.

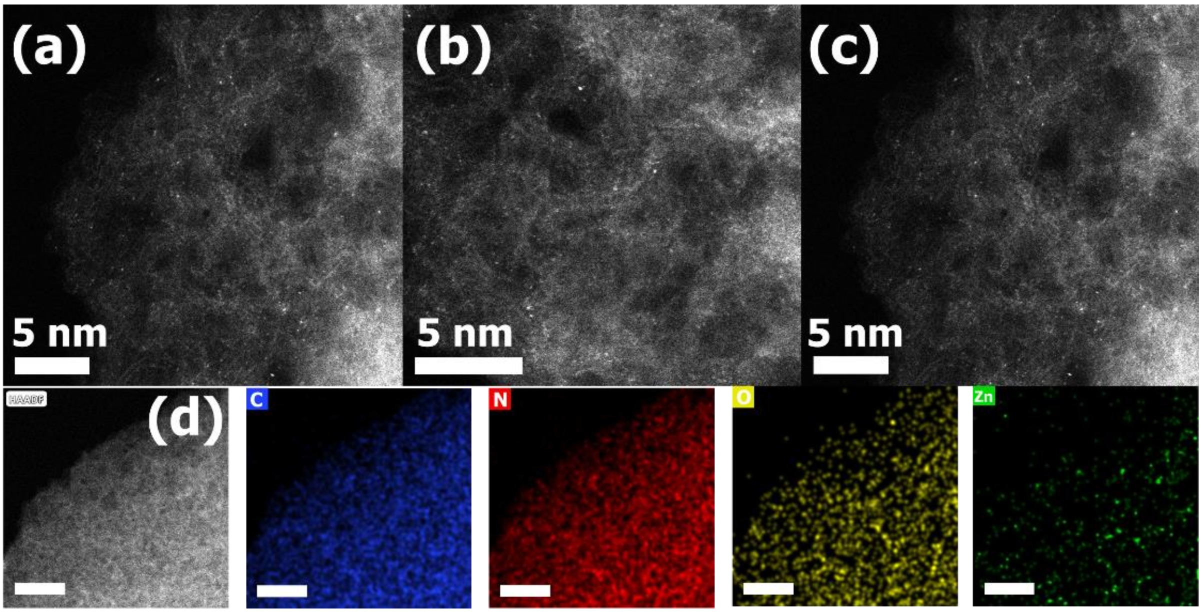

Figure S22. (a)-(c) Aberration-corrected high-angle annular dark-field scanning TEM image of post-ORR Zn-N_4-O. (d) Elemental mapping image of post-ORR Zn-N_4-O (scale bar, 30 nm).

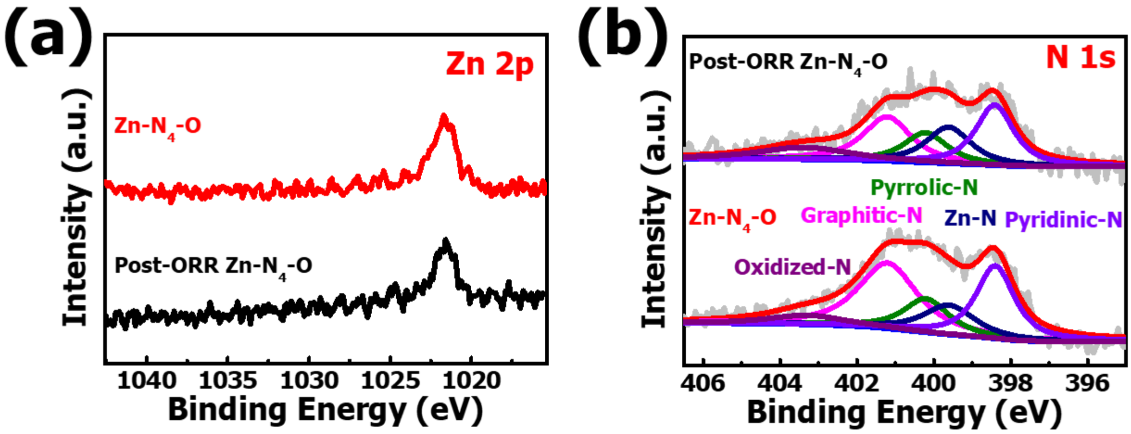

Figure S23. (a) Zn 2p and (b) N 1s spectra of Zn-N_4-O and post-ORR Zn-N_4-O.

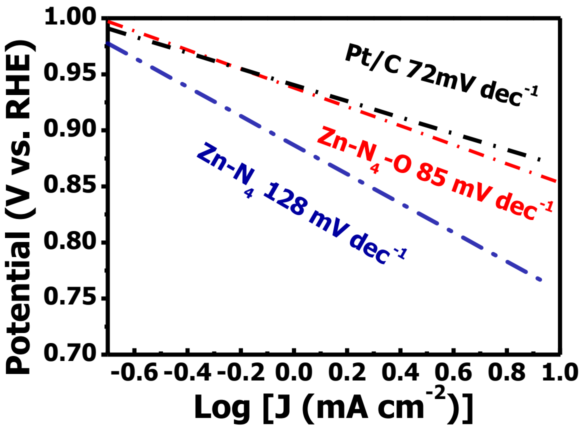

Figure S24. Tafel slope for Pt/C, Zn-N_4-O and Zn-N_4.

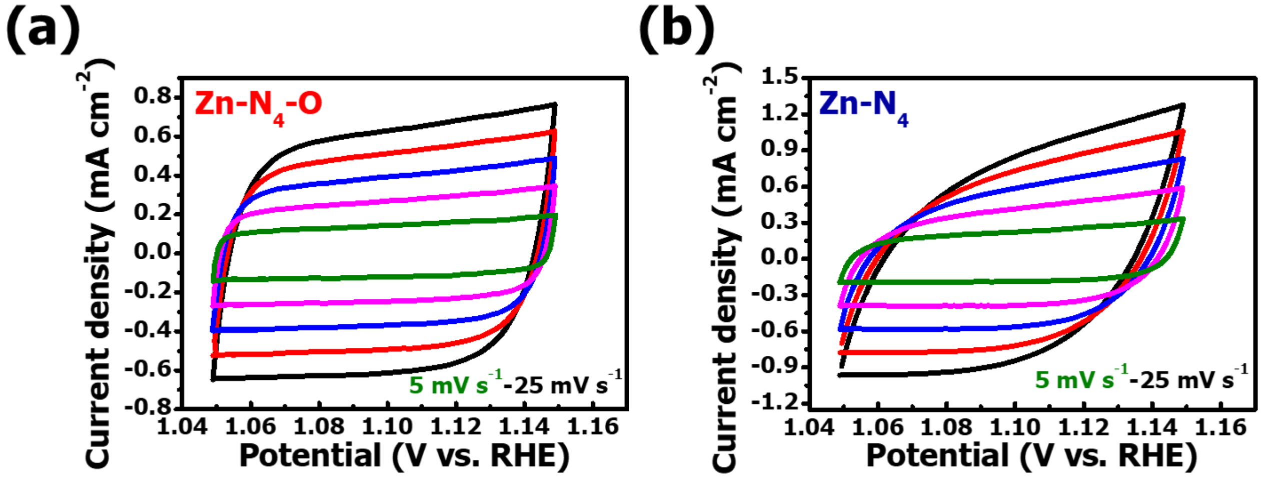

Figure S25. (a) Cyclic voltammograms for Zn-N_4-O in the region of 1.05-1.15 V vs RHE. (b) Cyclic voltammograms for Zn-N_4 in the region of 1.05-1.15 V vs RHE.

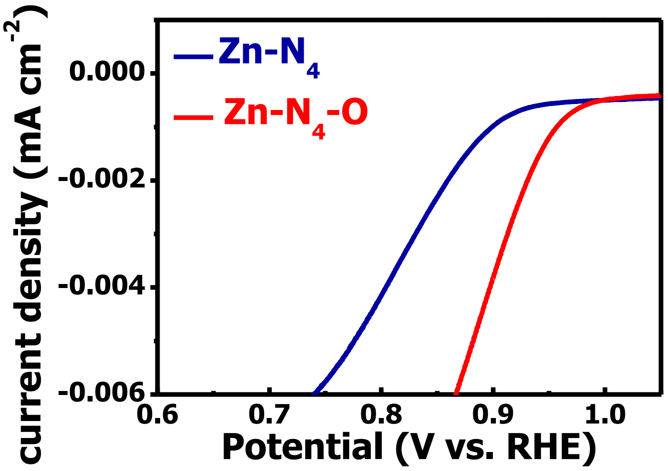

Figure S26. ECSA normalized ORR polarization curves of Zn-N_4-O and Zn-N_4 in 0.1 M KOH.

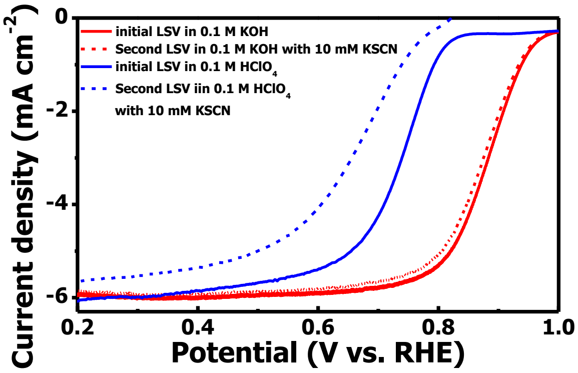

Figure S27. ORR polarization curves of Zn-N_4-O initially measured in O_2-saturated 0.1 M $HClO_4$ or 0.1 M KOH and the second curves measured after addition of 10 mM KSCN in the same electrolyte.

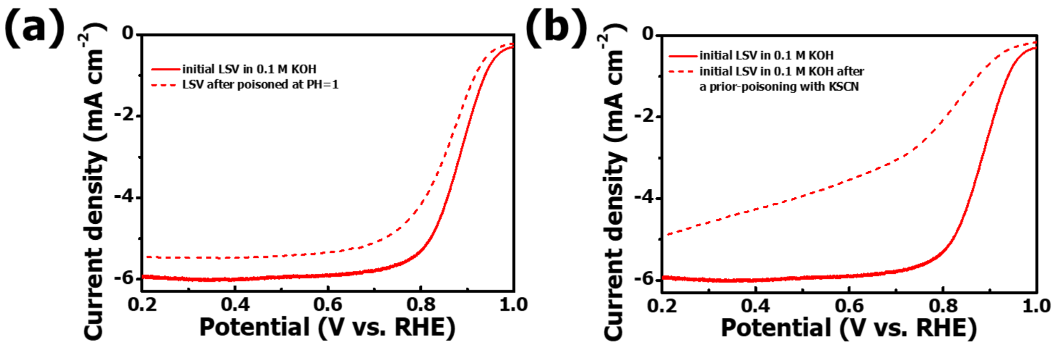

Figure S28. (a) ORR polarization curves of Zn-N_4-O in 0.1 M KOH after poisoned at PH=1 (0.1 M $HClO_4$ containing 10 mM KSCN). (b) ORR polarization curves of Zn-N4-O in 0.1 M KOH after prior-poisoning with KSCN.

We further employed more comprehensive poison experiments, which is an effective route to probe the active sites in single atom catalysts for ORR (J. Am. Chem. Soc. 2014, 136(31): 10882-10885; J. Phys. Chem. Lett. 2011, 2(4): 295-298). As shown in **Figure S27**, we conducted the KSCN poisoning measurements in both alkaline (0.1 M KOH) and acidic solutions (0.1 M $HClO_4$). After poisoning step, the activity of Zn-N_4-O was dramatically depressed in acidic electrolytes, but varied a little in alkaline electrolytes. In alkaline conditions, the hydroxyl and SCN^- will compete to adsorb on the metal sites (Adv. Sci. 2020, 7(12): 2000176). The large amounts of hydroxyl ligands can be formed on Zn sites and thus hiders the adsorption of SCN^-. Due to pH-dependent poisoning kinetics, the coordination of Zn-SCN is stable in the acidic condition but not in the alkaline condition (Nat. Commun. 2019, 10(1): 1278). Hence, according to previous works, we further conducted two poisoning experiments to avoid the influence of the OH^- and ensure the SCN^- adsorption on the Zn sites: (1) we first measured the ORR activity of Zn-N_4-O in O_2-saturated 0.1 M $HClO_4$ (PH=1) containing 10 mM SCN^- and subsequently transferred into O_2-saturated alkaline solution for a second LSV test (Nat. Commun. 2019, 10(1): 1278). (2) The catalysts ink was directly poisoned by SCN^- (prior-poisoning). Typically, 10 μL 2 M KSCN solution was added into the 200 μL catalysts ink for 10 minutes and the poisoned catalysts ink was tested in 0.1 M KOH (Adv. Sci. 2020, 7(12): 2000176). As shown in **Figure S28a-b**, after SCN^- adsorption on the Zn sites, the Zn-N_4-O exhibited significantly degenerated ORR activity in terms of obvious negative shift of the half-wave potential and notably reduced diffusion-limiting current density. The results corroborate that the Zn-N_4-O sites constitute the key catalytically active sites for ORR in alkaline conditions. As referee said, in situ spectroscopic studies would be useful to address the real active sites. The above poison experiments to some extent confirmed that Zn-related structure is the main active sites for ORR.

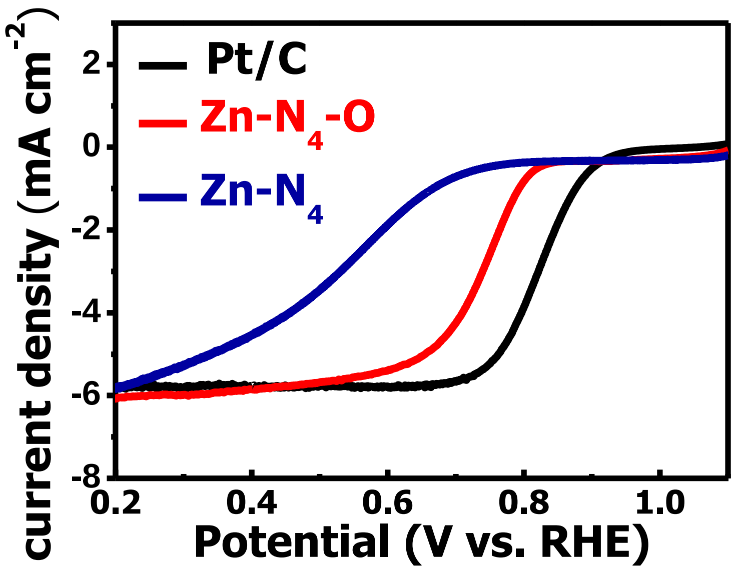

Figure S29. Polarization curves of Pt/C, Zn-N_4-O and Zn-N_4 in 0.1 M $HClO_4$.

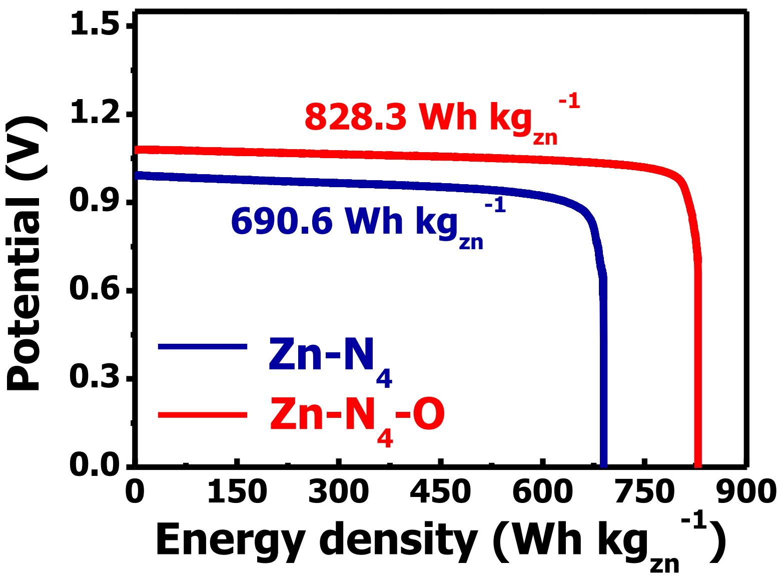

Figure S30. Energy density of the battery conducted with Zn-N_4-O and Zn-N_4.

Table S1. Zn content of Zn-N_4-O and Zn-N_4 determined by ICP-AES.

Sample	Zn content (wt.%)
Zn-N_4-O	0.43
Zn-N_4	2.5

Table S2. EXAFS fitting parameters at the **Zn** *K*-edge for various samples（S_0^2=0.897）

Sample	Shell	CN^a	R(Å)^b	σ^2(Å^2)^c	ΔE_0(eV)^d	R factor
Zn foil	Zn-Zn	12*	2.637±0.008	0.0114±0.0008	-1.8±1.3	0.0001
ZnO	Zn-O	4.0±0.2	1.958±0.005	0.0051±0.0007	2.4±0.8	0.0087
	Zn-Zn	12.0±0.5	3.222±0.005	0.0099±0.0005		
Zn-N_4-O	Zn-N/O	4.8±0.3	2.033±0.031	0.0088±0.0017	4.3±2.5	0.0002
Zn-N_4	Zn-N	4.3±0.5	1.974±0.008	0.0078±0.0014	2.6±1.2	0.0001

Table S3: The comparison of alkaline ORR activity of the recent earth-abundant catalysts and this work.

Catalyst	Catalyst loading (mg cm^{-2})	ORR half-wave potential (V vs. RHE)	Limit Current density (mA cm^{-2})	Reference
**N, Co-CNTs**	**0.4**	**0.884**	**5.9**	**This work**
FePhen@MOF-Ar NH_3	0.6	0.860	~6.0	Nat. Commun 2015, 6, 7343.
S, N-Fe/N/C-CNT	0.6	0.850	6.7	Angew. Chem. Int. Ed. 2017, 56, 610.
PFePc-I/C	0.25	0.948	~5.7	Adv. Energy Mater. 2022, 12, 2103588.
Zn/CoN-C	0.255	0.861	6.1	Angew. Chem. Int. Ed. 2019, 58, 2622-2626.
Co SAs/N-C(900)	0.408	0.881	~6	Angew. Chem. Int. Ed. 2016, 55, 10800.
FeSA-N-C	0.28	0.891	6.0	Angew.Chem. Int.Ed. 2018, 57,8525.
NC@Co-NGC DSNCs	0.4	0.82	~5.5	Adv. Mater. 2017, 29, 1700874.
CoN_4-O/MX	0.162	0.850	5.52	Adv. Funct. Mater. 2022, 2209499.
Zn-N-C-1	0.5	0.873	~5.0	Angew. Chem. Int. Ed. 2019, 58, 7035-7039.
FeN_4-O-NCR	0.3	0.942	5.5	Adv.Mater.2022, 34, 2202544.
Fe_2O_3/Fe_5C_2/Fe-N-C-3	0.25	0.870	5.34	Nano Lett. 2022, 22, 4879−4887.
Fe_{SA}-N-C	0.28	0.891	6	Angew.Chem.2018,57,8525.
Fe-ISA/SNC	0.51	0.896	5.5	Adv. Mater. 2018, 30, 1800588.
H-Co@FeCo/N/C	0.3	0.910	~5.7	Appl. Catal. B: Environ. 2020, 278, 119259.
PCN-FeCo/C	0.2	0.85	0.85	Adv. Mater. 2015, 27, 3431.
